# Solar‐Driven Hydrogen Peroxide Production via BiVO_4_‐Based Photocatalysts

**DOI:** 10.1002/advs.202407801

**Published:** 2024-12-08

**Authors:** Hui Ling Tan, Casandra Hui Teng Chai, Jerry Zhi Xiong Heng, Quyen Vu Thi, Xuelian Wu, Yun Hau Ng, Enyi Ye

**Affiliations:** ^1^ Institute of Sustainability for Chemicals Energy and Environment (ISCE2) Agency for Science Technology and Research (A*STAR) 1 Pesek Road, Jurong Island Singapore 627833 Singapore; ^2^ Institute of Materials Research and Engineering (IMRE) Agency for Science Technology and Research (A*STAR) 2 Fusionopolis Way Singapore 138634 Singapore; ^3^ School of Mechanical Engineering Chengdu University Chengdu 610106 China; ^4^ Chemical Engineering Program Physical Science and Engineering (PSE) Division King Abdullah University of Science and Technology (KAUST) Thuwal 23955‐6900 Saudi Arabia

**Keywords:** bismuth vanadate, hydrogen peroxide, photocatalysis, photoelectrocatalysis, powder suspension, oxygen reduction, water oxidation

## Abstract

Solar hydrogen peroxide (H_2_O_2_) production has garnered increased research interest owing to its safety, cost‐effectiveness, environmental friendliness, and sustainability. The synthesis of H_2_O_2_ relies mainly on renewable resources such as water, oxygen, and solar energy, resulting in minimal waste. Bismuth vanadate (BiVO_4_) stands out among various oxide semiconductors for selective H_2_O_2_ production under visible light via direct two‐electron oxygen reduction reaction (ORR) and two‐electron water oxidation reaction (WOR) pathways. Significant advancements have been achieved using BiVO_4_‐based materials in solar H_2_O_2_ production over the last decade. This review explores advancements in BiVO_4_‐based photocatalysts for H_2_O_2_ production, focusing on photocatalytic powder suspension (PS) and photoelectrochemical (PEC) systems, representing the main approaches for heterogenous artificial photosynthesis. An overview of fundamental principles, performance assessment methodologies, photocatalyst and photoelectrode development, and optimization of reaction conditions is provided. While diverse strategies, such as heterojunction, doping, crystal facet engineering, cocatalyst loading, and surface passivation, have proven effective in enhancing H_2_O_2_ generation, this review offers insights into their similar and distinct implementations within the PS and PEC systems. The challenges and future prospects in this field are also discussed to facilitate the rational design of high‐performing BiVO_4_‐based photocatalysts and photoelectrodes for H_2_O_2_ generation under visible light.

## Graphical abstract

This review systematically explores bismuth vanadate (BiVO_4_) photocatalysts for solar‐driven hydrogen peroxide (H_2_O_2_) production through artificial photosynthesis. It discusses the distinct functionalities of powder suspension (PS) and photoelectrochemical (PEC) configurations, highlighting their working principles for H_2_O_2_ generation and performance metrics. The paper further explores the optimization strategies for both systems through material and reaction parameter adjustments.

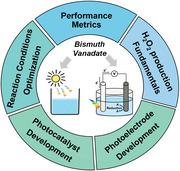

## 1 Introduction

Hydrogen peroxide (H_2_O_2_) is a versatile and green oxidant with vital applications across diverse industries, such as pulp and paper, textiles, chemical synthesis, wastewater treatment, electronics, and healthcare.^[^ ^1^ ^]^ Recognized as one of the world's 100 most important chemicals,^[^ ^2^ ^]^ H_2_O_2_ has garnered recent interest as a potential alternative energy carrier to hydrogen (H_2_), particularly for fuel cell applications due to its easy storability as an aqueous solution.^[^ ^3^ ^]^ While a H_2_O_2_ fuel cell produces a theoretical output voltage of 1.09 V, slightly lower than that of a conventional H_2_ fuel cell (1.23 V), the energy density of H_2_O_2_ (2.1 M J kg^−1^ for 60% aqueous H_2_O_2_) remains comparable to compressed H_2_ (3.5 M J kg^−1^).^[^ ^4^ ^]^ Compared to other liquid fuels such as methanol, H_2_O_2_ offers a more favorable zero‐emission alternative, fostering the transition toward carbon‐neutral energy sources. The global market demand for H_2_O_2_ is predicted to reach 5.7 million tons by 2027.^[^ ^5^ ^]^

Currently, ≈95% of global H_2_O_2_ production relies on the energy‐intensive multistage anthraquinone (AQ) oxidation process, which consumes significant volume of solvents and generates substantial wastewater effluents.^[^ ^1b^ ^]^ Due to efficiency considerations, the AQ process is typically centralized, resulting in the production of highly concentrated H_2_O_2_ (up to 70 wt%) to minimize transportation and distribution costs. However, many applications only require low‐concentration H_2_O_2_ solutions (1–10 wt%), necessitating dilution at the point of use.^[^ ^6^ ^]^ A more desirable approach would be decentralized, on‐site production of H_2_O_2_, achieved through the direct synthesis of H_2_O_2_ from oxygen (O_2_) and H_2_ using Pd or bimetallic Au‐Pd catalysts. However, safety concerns related to the explosive nature of the H_2_/O_2_ gas mixture impedes the implementation of this process.^[^ ^7^ ^]^ Artificial photosynthesis (AP) presents an appealing alternative for on‐site H_2_O_2_ synthesis. This technique utilizes a semiconductor photocatalyst to harness solar energy and drive the direct conversion of water (H_2_O) and O_2_ into H_2_O_2_ with minimal waste generation. By eliminating the need for fossil fuels and minimizing its environmental footprint, AP offers a safe, cost‐effective, eco‐friendly, and sustainable approach for H_2_O_2_ production.

Heterogeneous AP utilizes two main configurations: powder suspension (PS) and photoelectrochemical (PEC) systems. In PS systems, photocatalyst particles are suspended in an aqueous solution, while PEC systems involve depositing these particles onto a conductive substrate to form photoelectrodes, with either one or both electrodes being photoactive. Both PS and PEC systems harness light‐induced electron transfer reactions initiated by photocatalyst excitation, but their mechanisms for charge separation and transport differ significantly. While PEC systems spatially separate oxidation and reduction reactions on distinct electrodes, PS systems facilitate the redox reactions on the same photocatalyst particle surface, increasing the likelihood of charge recombination. As a result, optimizing photocatalyst performance may necessitate different strategies for PS and PEC systems. Our previous research demonstrated the contrasting effects of particle size on BiVO_4_ performance in photocatalytic PS and PEC water oxidation.^[^ ^8^ ^]^ Smaller particles enhanced charge transport and charge collection efficiency within BiVO_4_ photoelectrode, whereas larger particles benefited PS systems through improved charge separation facilitated by greater band bending and better crystallinity. Our recent review article further elaborated on the similarities and differences between these systems.^[^ ^9^ ^]^

To date, significant advancements have been made in developing organic semiconductors such as carbon nitride (C_3_N_4_) and Poly(9,9‐dioctylfluorene‐alt‐benzothiadiazole)/1‐[3‐(Methoxycarbonyl)propyl]‐1‐phenyl‐[6.6]C_61_ (PFBT/PCBM) polymer dots for H_2_O_2_ generation with commendable production yields of up to 3.76 mM h^−1^.^[^ ^10^ ^]^ However, their susceptibility to degradation due to poor chemical stability, particularly against hydroxyl radical (^•^OH) produced from H_2_O_2_ decomposition, remains a significant challenge limiting their practical applications.^[^ ^11^ ^]^ Consequently, inorganic metal oxide semiconductors, which generally offer relatively higher intrinsic chemical stability, are favored.^[^ ^12^ ^]^ Among the various metal oxides available for solar‐driven H_2_O_2_ generation, such as titanium dioxide (TiO_2_),^[^ ^13^ ^]^ tungsten oxide (WO_3_),^[^ ^14^ ^]^ and zinc oxide (ZnO),^[^ ^15^ ^]^ monoclinic scheelite bismuth vanadate (BiVO_4_) stands out as a highly promising photocatalyst. Monoclinic scheelite BiVO_4_ possesses a narrow bandgap of *≈*2.4 eV, enabling efficient utilization of the solar spectrum and possessing suitable band structures for two‐electron O_2_ reduction and H_2_O oxidation essential for selective H_2_O_2_ production. Furthermore, BiVO_4_ is predicted to facilitate easier charge extraction due to its much lighter effective masses of photogenerated carriers compared to other oxides such as TiO_2_ and In_2_O_3_.^[^ ^16^ ^]^ Nevertheless, the performance of BiVO_4_ for solar H_2_O_2_ generation is greatly hindered by its intrinsic shortcomings such as poor carrier mobility and poor charge separation.^[^ ^17^ ^]^ Additionally, the easy decomposition or disproportionation of H_2_O_2_ presents a challenge, resulting in typically low H_2_O_2_ yields by bare BiVO_4_. To address these challenges, numerous approaches, including material engineering and design (i.e., surface modification, crystal facet engineering, heterojunction formation, and doping) and optimization of reaction conditions, have been developed to enhance the visible‐light‐driven H_2_O_2_ generation by BiVO_4_‐based materials.

**Figure 1:**
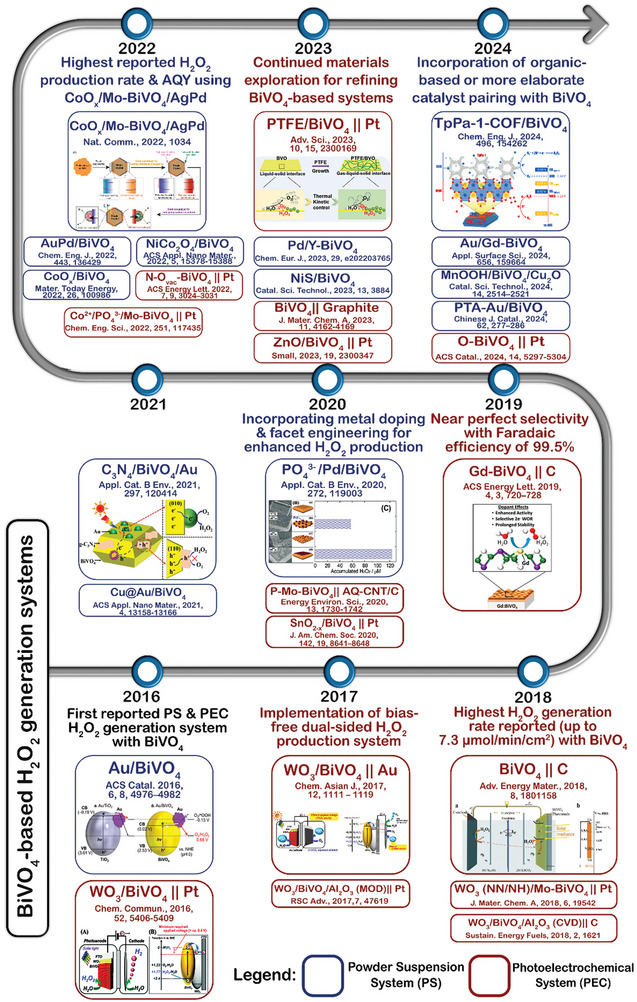
Chronology of BiVO_4_‐based materials developed for solar‐driven H_2_O_2_ production over the past decade.

While several reviews on solar‐driven H_2_O_2_ production have been published, they predominantly focus on the development of photocatalysts for either PEC^[^ ^18^ ^]^ or PS systems.^[^ ^10^, ^19^ ^]^ In contrast, reviews by Zeng et al.^[^ ^5^ ^]^ and Qu et al.^[^ ^20^ ^]^ provide insights into photocatalyst development for both PS and PEC systems, emphasizing reaction pathways and material engineering strategies, respectively. Although these reviews examine a broad range of photocatalysts, a comprehensive review that systematically compares the two main configurations of heterogeneous AP systems for H_2_O_2_ production, particularly focusing on a specific photocatalyst, is still lacking. A thorough understanding of the similarities and differences in the strategies employed to enhance the catalytic performance of the same material in these two distinct systems would be particularly valuable. Over the past decade, notable advancements have been made in the use of BiVO_4_‐based materials for solar H_2_O_2_ production (**Figure** **1**), underscoring the need for a detailed review of the strategies developed to enhance the performance of BiVO_4_‐based materials in both PS and PEC systems.

In this review, we elucidate the fundamental principles of H_2_O_2_ production in BiVO_4_‐based PS and PEC systems, dissecting the distinct reaction mechanisms. We assess the unique characteristics and challenges associated with each system, emphasizing the critical role of tailored material design and reaction optimization. Additionally, we provide a comprehensive overview of performance evaluation methods and strategies for optimizing H_2_O_2_ generation. By outlining the working principles and comparing the efficacy of both approaches, this review aims to inspire further research efforts toward the development of BiVO_4_‐based photocatalysts for sustainable solar‐driven H_2_O_2_ production under visible light. While the paper primarily focuses on BiVO_4_‐based materials, the established principles governing H_2_O_2_ production, along with the detailed performance evaluation methodologies and material design strategies outlined here can serve as valuable frameworks for researchers exploring alternative photocatalysts for AP systems.

## 2 Fundamentals of Solar‐Driven H_2_O_2_ Production over BiVO_4_

When a semiconductor photocatalyst absorbs photons with energy greater than its bandgap, electrons are excited to the conduction band (CB), creating vacancies known as holes in the valence band (VB). These photogenerated electron–hole pairs drive the production of solar H_2_O_2_ on the surface of the photocatalyst via O_2_ reduction and/or H_2_O oxidation reactions (ORR and WOR, respectively).^[^ ^5^, ^20^ ^]^ This section delves into the reaction pathways for H_2_O_2_ production using BiVO_4_ in PS and PEC systems.

### 2.1 Powder Suspension (PS) System

**Figure 2:**
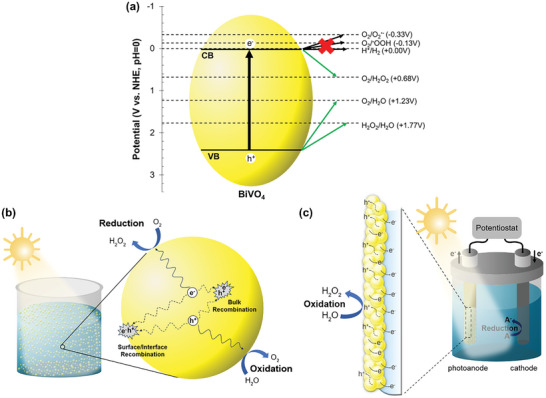
Schematic representation of H_2_O_2_ production on BiVO_4_. a) Energy level diagram illustrating the selective reduction of O_2_ to H_2_O_2_ on BiVO_4_ photocatalyst. The conduction band (CB) potential disfavors H_2_ evolution and one‐electron O_2_ reduction, promoting the two‐electron pathway to H_2_O_2_ while enabling water oxidation via valence band (VB) holes. b,c) Schematic of b) a photocatalytic (PS) system and c) a photoelectrochemical (PEC) system for H_2_O_2_ production, highlighting the distinct mechanisms of charge separation, transport, and transfer processes in the respective configurations. Note that A = electron acceptor; A^−^ = reduced electron acceptor.

For PS system, the primary redox reactions involve a direct two‐electron reduction of O_2_ to H_2_O_2_ (Equation 1) and a four‐electron oxidation of H_2_O to O_2_ (Equation 2). This indicates that H_2_O_2_ can be generated from O_2_ and H_2_O with an overall four‐electron transfer (Equation 3). However, undesirable side reactions can occur, including the ORR competing with one‐electron reduction pathways for reactive oxygen species (ROS) generation (e.g., ^•^O_2_^−^ and ^•^OOH) and H_2_O reduction for H_2_ evolution. These side reactions reduce the semiconductor's selectivity for H_2_O_2_ production. Although H_2_O_2_ can also be formed through an indirect stepwise one‐electron ORR, where ^•^O_2_^−^ formed from O_2_ reduction is further reduced to H_2_O_2_, the selectively for H_2_O_2_ production is lower compared to the direct two‐electron pathway. Among metal oxides materials, BiVO_4_, characterized by a CB with a weak reducing potential at *≈* +0.02 V versus NHE, shows promise in inhibiting these major side reactions, as depicted in **Figure** **2**a. This makes BiVO_4_ a highly favorable material for achieving selective and efficient H_2_O_2_ generation in the PS system.

(1) $$\begin{array}{c} O_{2}+2H^{+}+2e^{-}\to H_{2}O_{2}\;\;\;E^{\circ }\left(O_{2}/H_{2}O_{2}\right)\\ =+0.68V\;\mathrm{versus}\;\mathrm{NHE} \end{array}$$

(2) $$\begin{array}{c} 2H_{2}O\to O_{2}+4H^{+}+4e^{-}\;\;\;\;\;\;E^{\circ }\left(O_{2}/H_{2}O\right)\\ =+1.23V\;\;\mathrm{versus}\;\;\mathrm{NHE} \end{array}$$

(3) $$\begin{array}{c} 2\;\mathrm{Equation}\left(1\right)+\mathrm{Equation}\left(2\right):2H_{2}O+O_{2}\\ \to 2H_{2}O_{2}\left(\mathrm{Four}-\mathrm{electron}\mathrm{process}\right) \end{array}$$

Nevertheless, the photocatalytic H_2_O_2_ production process is hampered by H_2_O_2_ decomposition, induced by photogenerated charge carriers in the presence of transition metals or organic compounds. H_2_O_2_ can either be reduced by the photogenerated electrons to form ^•^OH (Equation 4), oxidized by the photogenerated holes to form ^•^O_2_^−^ (Equation 5), or disproportionate to form H_2_O and O_2_.^[^ ^21^ ^]^ Additionally, H_2_O_2_ can easily decompose in the presence of ultraviolet light irradiation or heat stimuluses.^[^ ^22^ ^]^

(4) $$H_{2}O_{2}+e^{-}\to^{•}\mathrm{OH}+OH^{-}\;$$

(5) $$H_{2}O_{2}+h^{+}\to^{•}{O_{2}}^{-}+2H^{+\;\;}$$

With semiconductor particles freely suspended in an aqueous medium, both reduction and oxidation reactions take place simultaneously on the surface of the same particle in the PS system. As photogenerated electrons and holes have to diffuse from the bulk to the surface, they become susceptible to recombination through radiative or nonradiative processes occurring in the bulk, at the interface, and at the surface (Figure 2b).^[^ ^23^ ^]^ These processes diminish the number of usable charge carriers for redox reactions and deteriorate the photocatalytic performance of a semiconductor, highlighting the significance of charge separation in the PS system.

### 2.2 Photoelectrochemical (PEC) System

In contrast, a PEC system utilizes photoelectrode(s) made by immobilizing the photocatalyst on conducting substrate(s) such as fluorine‐doped tin oxide‐coated glass (FTO). Photogenerated electrons are drawn from the photoanode to the counter electrode with the assistance of an external bias, enabling redox reactions to occur independently at each electrode. This eliminates the necessity for efficient charge separation within the photocatalyst. However, photogenerated electrons have to transport through the photocatalyst particles to the back substrate for effective collection and transfer of electrons through the external circuit, whereas holes move to the interface between the photoanode and the electrolyte for oxidation reaction (Figure 2c). This indicates charge transport as a more critical factor in the PEC system compared to charge separation efficiency.^[^ ^8^ ^]^

Similar to the PS system, H_2_O_2_ can be synthesized through cathodic direct two‐electron ORR (Equation 1) with H_2_O oxidized at the anode (Equation 2). However, PEC offers another attractive route: anodic two‐electron WOR (Equation 6). This approach is highly favored as it can be coupled with H_2_ generation from H_2_O reduction (Equation 7) at the cathode, resulting in the simultaneous production of H_2_O_2_ and H_2_ (Equation 8)—a highly sought‐after low‐carbon energy carrier.^[^ ^24^ ^]^

(6) $$\begin{array}{c} 2H_{2}O\to H_{2}O_{2}+2H^{+}+2e^{-}\;\;\;\;\;\;\;E^{\circ }\left(H_{2}O_{2}/H_{2}O\right)\\ =+1.77V\;\;\mathrm{versus}\;\mathrm{NHE} \end{array}$$

(7) $$2H^{+}+2e^{-}\to H_{2}\;\;\;\;\;\;E^{\circ }\left(H^{+}/H_{2}\right)=+0.00V\;\;\mathrm{versus}\;\;\mathrm{NHE}$$

(8) $$\mathrm{Equation}\left(6\right)+\mathrm{Equation}\left(7\right):2H_{2}O\to H_{2}O_{2}+H_{2}$$

Nonetheless, the anodic two‐electron WOR for H_2_O_2_ production has two major challenges. First, the WOR to O_2_ is thermodynamically favored over H_2_O_2_ production due to a lower thermodynamic barrier. Second, the produced H_2_O_2_ is susceptible to further oxidation back to O_2_ on the anode (Equation 1). Suppressing these competitive reactions is imperative in achieving high H_2_O_2_ production selectivity and accumulation concentration.

**Figure 3:**
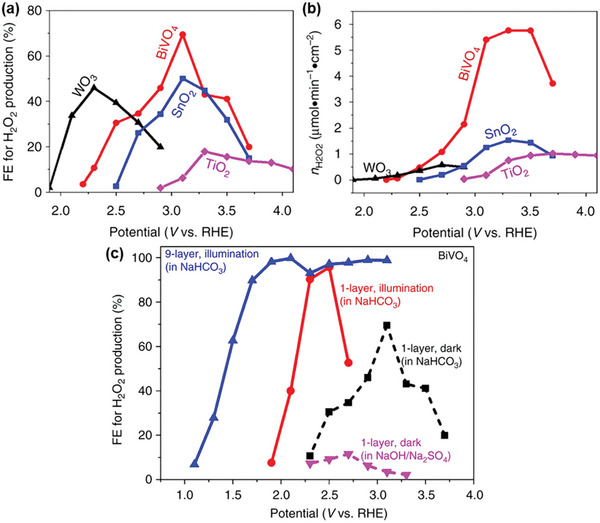
(Photo)electrochemical performance of metal oxide photoanodes for H_2_O_2_ production indicating BiVO_4_ as the best‐performing anode material. a) Faraday efficiency (FE) for H_2_O_2_ production and b) H_2_O_2_ yield during anodic H_2_O oxidation over various metal oxide photoanodes as a function of applied potential in the dark. c) FE for H_2_O_2_ production of BiVO_4_ under different experimental conditions, including various electrolyte types (NaHCO_3_, Na_2_SO_4_) and exposure to light or darkness. Reproduced with permission.^[^ ^24b^ ^]^ Copyright 2017, Springer Nature.

As an n‐type semiconductor with a suitable VB potential for H_2_O_2_/H_2_O oxidation (depicted in Figure 2a), BiVO_4_ stands out as an ideal photoanode material for PEC H_2_O_2_ production via the two‐electron WOR pathway. While its function for water oxidation to O_2_ has been extensively studied,^[^ ^25^ ^]^ its potential for H_2_O_2_ production is scantly explored. Shi et al. assessed various metal oxides for their ability to electrochemically produce H_2_O_2_ through anodic H_2_O oxidation.^[^ ^24b^ ^]^ Density functional theory (DFT) calculations and experimental measurements determined an onset potential sequence of WO_3_ < BiVO_4_ < SnO_2_ < TiO_2_, and BiVO_4_ was experimentally validated as the most promising anode candidate for electrochemical and PEC water oxidation to form H_2_O_2_. Under dark conditions, BiVO_4_ produced the highest Faraday efficiency for H_2_O_2_ production (FE(H_2_O_2_)) of 70% at 3.1 V versus RHE (**Figure** **3**a) and the highest amount of H_2_O_2_ (Figure 3b). When light was introduced, optimizing the electrolyte and BiVO_4_ thickness boosted the FE(H_2_O_2_) to 98% and decreased its onset potential from 2.2 V under dark conditions to *≈*1.1 V (Figure 3c). This aligns with other research screening different metal oxides (i.e., CoO, WO_3_, La_2_O_3_, Nb_2_O_5_, Al_2_O_3_, TiO_2_, ZrO_2_, V_2_O_5_, Bi_2_O_3_, and BiVO_4_) for electrochemical H_2_O_2_ production, where BiVO_4_ was identified as one of the best‐performing anode materials.^[^ ^24a^ ^]^

Alternatively, coupling anodic two‐electron WOR with cathodic two‐electron ORR leads to a more energetically efficient two‐electron dual‐channel pathway for H_2_O_2_ production (Equation 9).

(9) $$\begin{array}{c} \mathrm{Equation}\left(1\right)+\mathrm{Equation}\left(6\right):2H_{2}O+O_{2}\\ \to 2H_{2}O_{2}\left(\mathrm{Two}-\mathrm{electron}\mathrm{process}\right)\; \end{array}$$

Leveraging on BiVO_4_’s favorable CB and VB potentials which straddle the redox potentials of H_2_O_2_ (as illustrated in Figure 2a), a PEC system with a BiVO_4_ photoanode holds promise for achieving the highly coveted dual‐side H_2_O_2_ production at both the photoanode and cathode. This configuration eliminates the need for an external bias, offering a more efficient and potentially simpler system for H_2_O_2_ production.

## 3 Powder Suspension (PS) System

**Table** **1** presents an overview of the reported photocatalytic performance of BiVO_4_‐based materials for H_2_O_2_ generation, particularly in a PS system. While BiVO_4_’s band potentials are suitable for driving photocatalytic H_2_O_2_ production from two‐electron ORR and four‐electron WOR, its bare form exhibits a negligible H_2_O_2_ production rate, often below 1 µM h^−1^. This limitation is primarily attributed to inherent material constraints, such as rapid charge recombination, slow hole mobility, and lack of surface active sites for ORR. However, significant advancements have been achieved over the past decade, with modified BiVO_4_‐based materials demonstrating improvements of 1–3 orders of magnitude compared to bare BiVO_4_. This section delves into various modification methods and reaction conditions used in the PS system, along with mechanisms behind this performance enhancement. To establish a common ground for understanding material development, we first describe the experimental characterization methods used to evaluate the H_2_O_2_ generation in PS systems.

**Table 1:** Summary of photocatalytic H_2_O_2_ production via a PS system using BiVO_4_‐based materials dispersed in an O_2_‐saturated aqueous solution.

Photocatalyst	Faceted BiVO_4_	Experimental Conditions				H_2_O_2_ Production Rate [µM h^−1^]^a)^	Enhancement in H_2_O_2_ Production Rate Compared to Unmodified BiVO_4_ ^b)^	AQY [%] at 420 nm	Ref
		Light	Sacrificial Reagent	Buffer Solution	Temperature Control [°C]				
Au/BiVO_4_	No	λ > 420 nm	No	–	25	4	8	0.24	[26]
Pd/BiVO_4_	Yes	λ > 420 nm	Yes	–	Ice bath	170	3400	–	[27]
Phosphate ion coated Pd/BiVO_4_	Yes	λ > 420 nm	Yes	–	Ice bath	300	6000	–	[27]
Cu@Au core–shell /BiVO_4_	Yes	λ = 420 nm	Yes	–	–	30	91	0.88	[28]
AuPd/BiVO_4_	Yes	λ = 420 nm	No	citrate buffer (pH 3)	20	1145	2	11.38	[29]
NiS/BiVO_4_	Yes	λ = 420 nm	No	citrate buffer	–	488	87	4.8	[30]
CoO*_x_*/BiVO_4_	Yes	λ > 420 nm	Yes	–	–	2	2	–	[31]
CoO*_x_*/Mo‐doped BiVO_4_/Pd	Yes	AM 1.5G	No	phosphate buffer (pH 7.4)	12±0.5	1425	14250	5.8	[32]
CoO*_x_*/Mo‐doped BiVO_4_/ core–shell AgPd	Yes	AM 1.5G	No	–	12±0.5	9700	–	13.1	[33]
Pd/Y‐doped BiVO_4_	No	AM 1.5G	Yes	phosphate buffer (pH 7)	5±0.5	15	4	–	[34]
Au/Gd‐doped BiVO_4_	Yes	λ = 420 nm	Yes	–	20	1035	200	7.44	[35]
C_3_N_4_/BiVO_4_/Au	Yes	λ = 420 nm	No	citrate buffer (pH 3)	20	676	151	6.7	[36]
NiCo_2_O_4_/BiVO_4_	No	λ > 420 nm	No	–	–	390	16	3.27	[37]
Au/BiVO_4_ QD/PTA	No	λ > 420 nm	No	–	25	219	11	–	[38]
MnOOH/BiVO_4_/Cu_2_O	Yes	λ = 420 nm	No	phosphate buffer	20	112	16	–	[39]
TpPa‐1‐COF/BiVO_4_	Yes	λ > 420 nm	No	–	–	1205	4	7.2	[40]

^a)^ Estimated based on the reported H_2_O_2_ concentration at the given reaction duration;

^b)^ Estimated as fold increase relative to the unmodified BiVO_4_ using the formula $\frac{H_{2}O_{2}\;\mathrm{production}\;\mathrm{rate}\;\mathrm{of}\;\mathrm{modified}\;\mathrm{BiV}O_{4}}{H_{2}O_{2}\;\mathrm{production}\;\mathrm{rate}\;\mathrm{of}\;\mathrm{unmodified}\;\mathrm{BiV}O_{4}}$.

### 3.1 Evaluation of Photocatalytic Performance for H_2_O_2_ Generation

In photocatalytic studies using PS system, material performance is evaluated by activity, stability, and solar conversion efficiency. For H_2_O_2_ generation, the reactor setup involves dispersing the photocatalyst particles in an O_2_‐saturated aqueous solution under light irradiation. The temperature is typically controlled at room temperature or lower to minimize the thermal decomposition of H_2_O_2_. Since H_2_O oxidation to O_2_ is slow, alcohols such as methanol or ethanol can be added as hole scavengers (also termed as sacrificial reagents) to minimize charge recombination and promote H_2_O_2_ production. Photocatalytic activity is evaluated by the amount of H_2_O_2_ produced upon light exposure. The produced H_2_O_2_, typically in the liquid phase, can be detected and quantified using various analytical techniques (**Table** **2**). These techniques often involve oxidizing or reducing H_2_O_2_ to create a colored compound with specific light absorption or emission wavelengths. Ultraviolet–visible (UV–vis) spectroscopy is commonly employed in tandem with titrimetric or colorimetric techniques due to its high accuracy and reduced susceptibility to human error. This allows for precise quantification based on the absolute absorbance values of the colored compound.

**Table 2:** Summary of analytical techniques reported in the literature for quantifying H_2_O_2_ in the liquid phase using BiVO_4_‐based materials.

Analytical Technique	Description	Pros	Cons	Ref
Titrimetric	a) Titrate H_2_O_2_ with potassium permanganate	High accuracy	Labor intensive	[26]
Colorimetric + Spectrophotometric	a) Monitor Fe^2+^ color change to Fe^3+^ via reaction with H_2_O_2_ in acidic condition based on the following equation: Fe^2+^ + H_2_O_2_ → Fe^3+^ + 2OH^−^ Measure Fe^3+^ formation with UV–vis spectrophotometer at 305/330 nm absorbance wavelengths.	High accuracy and sensitivity	Linear calibration is required to obtain a standard curve	[22, 27, 41]
	b) Monitor the formation of the yellow‐colored epoxide product resulting from the reaction between H_2_O_2_ and potassium titanium oxide oxalate dihydrate (K_2_TiO(C_2_O_4_)_2_·2H_2_O) using a UV–vis spectrophotometer at an absorbance wavelength of 385 nm.			[28, 29, 36]
	c) Monitor the formation of copper (I)‐DMP complex resulting from the reduction of Cu^2+^ by H_2_O_2_ in the presence of excess 2,9‐dimethyl‐1,10‐phenanthroline (DMP) using a UV–vis spectrophotometer at an absorbance wavelength of 454 nm. The reaction can be represented as: 2Cu^2+^ + 2DMP + H_2_O_2_ → 2Cu(DMP)_2_^+^ + O_2_ + 2H^+^			[37, 42]
	d) Monitor the formation of the pink‐colored N,N‐diethyl‐1,4‐phenylene‐diamine (DPD) radical cation resulting from the reaction between DPD and peroxidase using a UV–vis spectrophotometer at an absorbance wavelength of 551 nm.			[43]
	e) Monitor the formation of the dark greenish carbonatocobaltate complex (Co(CO_3_)_3_^3−^) resulting from the reaction between H_2_O_2_ and Co^2+^ in the presence of bicarbonate using a UV–vis spectrophotometer at an absorbance wavelength of 257 nm.			[44]
Titrimetric + Spectrophotometric	a) Monitor the formation of the I^3−^ product resulting from the reaction between H_2_O_2_ and potassium iodide, catalyzed by ammonium molybdate ((NH_4_)_6_Mo_7_O_24_.4H_2_O), using a UV–vis spectrophotometer at an absorbance wavelength of 352 nm.	High accuracy and sensitivity	Linear calibration is required to obtain a standard curve	[31]
HPLC colorimetric	a) Reaction of H_2_O_2_ with ampliflu red in the presence of horseradish peroxidase to produce resorufin detected using HPLC‐DAD at 560 nm.	Rapid and selective	High cost and equipment requirements	[32, 33]

The amount of H_2_O_2_ produced and accumulated over a specific reaction time (i.e., production) is typically reported in concentration units such as µmol l^−1^ or µM. However, comparing studies solely based on H_2_O_2_ concentration can be challenging due to varying experimental conditions such as solvent volume, mass of photocatalyst, and reaction duration. To address this, calculating the production rate or productivity as µmol g^−1^ l^−1^ h^−1^ eliminates these variables and serve as a more suitable metric for comparison. It is important to note that high productivity does not always correspond to high production, as they are inversely correlated. Productivity may decrease over time while total H_2_O_2_ production increases.^[^ ^21^ ^]^

Photocatalytic H_2_O_2_ production involves two competing pathways: H_2_O_2_ formation and decomposition. In an O_2_‐saturated solution, the formation rate (*k*_f_) is assumed to be constant (zero‐order kinetics), while the decomposition rate (*k*_d_) is proportional to existing H_2_O_2_ concentration (first‐order kinetics).^[^ ^45^ ^]^ The rate constant for H_2_O_2_ (*k*_f_) is an important parameter as a higher *k*_f_ signifies a faster rate of H_2_O_2_ production by the photocatalyst.

(10) $$\left[H_{2}O_{2}\right]=\frac{k_{f}}{k_{d}}\;\left(1-\mathrm{ex}p^{\left(-k_{d}t\right)}\right)$$

Equation (10) expresses the relationship between H_2_O_2_ concentration ([H_2_O_2_]), the rate constants *k*_f_ and *k*_d_, and the illumination time (*t*), and allows for the estimation of *k*_f_ based on the known *k*_d_ value. *k*_d_ can be determined by a first‐order reaction kinetic model through a dedicated H_2_O_2_ decomposition experiment. The experiment involves exposing a known amount of commercial H_2_O_2_ solution to the studied photocatalyst under light irradiation and monitoring the decrease in H_2_O_2_ concentration over time.

The stability of a photocatalyst in the PS system is commonly assessed by recycling the material over a series of activity tests. Typically, the spent photocatalyst is recovered from one activity test and reused for subsequent tests under identical experimental conditions, repeated multiple times. One main challenge lies in fully retrieving the dispersed photocatalyst, leading to potential material loss. While there is no single standardized metric for comparing stability in PS systems, a stable photocatalyst should maintain comparable activity levels over multiple cycles. Additionally, characterization techniques such as electron microscopy (e.g., scanning electron microscopy (SEM) and transmission electron microscopy (TEM)), X‐ray diffraction (XRD), and X‐ray photoelectron spectroscopy (XPS) can be used alongside recycling tests for a more comprehensive evaluation of a photocatalyst's stability. Characterization of the photocatalyst before and after an activity test enables detection of potential changes in its physicochemical properties.

Considering that light scattering commonly occurs in PS systems, the efficiency of the photocatalyst to convert absorbed photons into desired chemical products can be evaluated by apparent quantum yield or efficiency (AQY/AQE) and solar‐to‐chemical conversion (SCC) efficiency. AQY/AQE measures the ratio of the number of electrons involved in a reaction under a particular wavelength irradiation to the total incident photons, while SCC efficiency refers to the effectiveness of the photosystem in converting solar energy into chemical energy. For H_2_O_2_ production, the AQY/AQE (Equation 11) and solar‐to‐H_2_O_2_ (STH) (Equation 12) for photocatalytic H_2_O_2_ production can be calculated as follows:

(11) $$\begin{array}{ccc} \mathrm{AQY}\;\mathrm{or}\;\mathrm{AQE}\;\;\left(\%\right) & & =\frac{2\times \mathrm{Amount}\;\mathrm{of}\;H_{2}O_{2}\;\mathrm{generated}}{\mathrm{Number}\;\mathrm{of}\;\mathrm{incident}\;\mathrm{photons}}\;\times \;100\\ & & =\frac{2\times {\left[H_{2}O_{2}\right]}_{t}\times V\times N_{a}}{P\times A\times t\times \frac{\lambda }{hc}}\times 100 \end{array}$$

(12) $$\begin{array}{ccc} \mathrm{STH}\left(\%\right) & & =\frac{\mathrm{Energy}\;\mathrm{output}\;\mathrm{as}\;H_{2}O_{2}}{\mathrm{Energy}\;\mathrm{of}\;\mathrm{incident}\;\mathrm{light}}\\ & & =\frac{\Delta G\left(H_{2}O_{2}\right)\times {\left[H_{2}O_{2}\right]}_{t}\times V}{P\times A\times t}\times 100 \end{array}$$

where [H_2_O_2_] is the H_2_O_2_ concentration at a specify reaction time *t*, *V* is the volume of suspension, *N*_a_ is the Avogadro constant (6.02 × 10^23^ mol^−1^), *P* is the intensity of incident monochromatic light (W cm^−2^), *A* is the irradiation area (cm^2^), *λ* is the wavelength, *h* is the Planck's constant (6.626 × 10^−34^ J s^−1^), *c* is the speed of light in vacuum (3 × 10^8^ m s^−1^), and Δ*G*(H_2_O_2_) is the free energy for H_2_O_2_ formation (117 kJ mol^−1^).

Based on the band structure of BiVO_4_ (Section 2.1.), the photocatalytic production of H_2_O_2_ in a PS system theoretically involves four‐electron oxidation of H_2_O to O_2_ and two‐electron reduction of O_2_ to H_2_O_2_ reactions. While the oxidative half‐reaction can be validated using AgNO_3_ as the electron scavenger, confirming the two‐electron ORR is more intricate due to competing one‐electron reactions generating reactive oxygen species such as ^•^O_2_^−^ and ^•^OOH. The following two techniques can verify the two‐electron ORR half‐reaction pathway:

i) Electron Paramagnetic Resonance (EPR) analysis: This technique detects the generation of ^•^O_2_^−^ and ^•^OOH radicals using a radical scavenger such as 5,5‐dimethyl‐1‐pyrroline‐N‐oxide (DMPO). The absence of these radicals suggests a two‐electron ORR pathway.
ii) Koutecky–Levich plot analysis: This method utilizes a rotating disk electrode to determine the number of electrons (n) involved in ORR. The linear region of the plot follows the equation:
(13) $$J^{-1}={J_{k}}^{-1}+B^{-1}\omega^{-1/2}$$

where *J* is the measured current density (A cm−^2^), *J_k_* is the kinetic current density (A cm−^2^), *ω* is the angular velocity of the disk (rad s^−1^), and *B* is the Levich constant defined as follows:

(14) $$B=0.62nF\nu^{-1/6}CD^{2/3}$$

where *F* is the Faraday constant (96485 C mol^−1^), *ν* is the kinematic viscosity of water (0.01 cm^2^ s^‒1^), *C* is saturated concentration of O_2_ in solution (1.3 × 10^−6^ mol cm−^3^), and *D* is the diffusion coefficient of O_2_ in water (2.7 × 10^−5^ cm^2^ s^−1^).

In this analysis, the slope of the Koutecky–Levich plot (*S*) corresponds to *B* ^−1^. Therefore, the number of electrons (*n*) can be estimated using the following equation:

(15) $$n=\frac{1}{0.62SFv^{-1/6}CD^{2/3}}$$

### 3.2 Strategies for Enhanced Photocatalytic H_2_O_2_ Production

**Figure 4:**
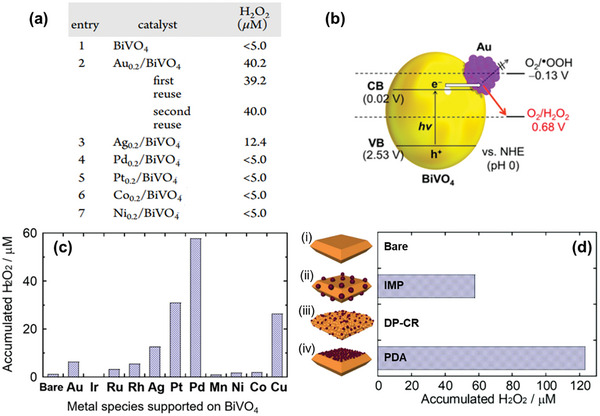
The performance of different metals as cocatalyst to improve the photocatalytic H_2_O_2_ production on BiVO_4_. a) H_2_O_2_ production of bare BiVO_4_ and various metal‐loaded BiVO_4_ photocatalysts under visible‐light irradiation (λ > 420 nm) in pure water for 10 h. b) Band potentials of Au/BiVO_4_ compared to the O_2_ reduction potential. Reproduced with permission.^[^ ^26^ ^]^ Copyright 2016, American Chemical Society. c) H_2_O_2_ production performances of BiVO_4_ loaded with different metal cocatalysts. d) Comparison of H_2_O_2_ production performances of bare BiVO_4_ and Pd‐loaded BiVO_4_ prepared by different methods: i) impregnation (IMP), ii) deposition–precipitation and chemical reduction (DP‐CR), and iii) photodeposition (PDA), respectively, in aqueous methanol solution under visible‐light irradiation (λ > 420 nm) for 2 h. Reproduced with permission.^[^ ^27^ ^]^ Copyright 2020, Elsevier.

One commonly employed strategy to improve photocatalyst performance involves loading a cocatalyst onto the photocatalyst surface. These cocatalysts provide active sites and tune interfacial energetics by promoting charge separation and enhancing surface reaction kinetics for the targeted reactions. Hirakawa and coworkers were the first to successfully demonstrate an all‐inorganic system for photocatalytic H_2_O_2_ synthesis using Au‐loaded BiVO_4_. Their work achieved significant enhancement in H_2_O_2_ generation directly from H_2_O and O_2_ (40.2 µM H_2_O_2_ after 10 h of visible‐light irradiation), compared to negligible amounts (<5.0 µM) with bare BiVO_4_.^[^ ^26^ ^]^ Notably, among various metals tested (Ag, Pd, Pt, Co, and Ni), only Au effectively promoted H_2_O_2_ formation on BiVO_4_ (**Figure** **4**a). While H_2_O oxidation is suggested as the rate‐determining step, the selectivity toward the two‐electron reduction pathway is attributed to BiVO_4_’s CB potential being more positive than the potential for one‐electron reduction of O_2_, therefore, suppressing the undesired one‐electron pathway (Figure 4b).

In contrast, a study conducted by Fuku et al. suggests Pd as the most effective metal cocatalyst for promoting H_2_O_2_ production from O_2_, using methanol as an electron donor and BiVO_4_ as the photocatalyst.^[^ ^27^ ^]^ Most metal cocatalysts, apart from Ir and Mn, improved H_2_O_2_ yield compared to bare BiVO_4_, with Pd‐loaded BiVO_4_ showing a remarkable 9.3‐fold improvement over Au‐loaded BiVO_4_ (Figure 4c). This superior performance was ascribed to Pd's lower overpotential for O_2_ electrochemical reduction than Au, and its higher selectivity for the two‐electron reduction of O_2_ compared to Pt. Interestingly, the selective deposition of Pd on the {010} facets of BiVO_4_ (via photodeposition) led to the best performance (Figure 4d). This aligns with the seminal work by Li et al., where {010} and {110} facets were identified as reduction and oxidation functional sites of BiVO_4_, respectively, due to spatial separation of photogenerated electrons and holes on the two facets.^[^ ^46^ ^]^ Given that Pd is the reduction cocatalyst that promotes O_2_ reduction to H_2_O_2_, its deposition on the electron‐accumulated {010} facet is strategically beneficial for H_2_O_2_ production. Such a rational loading of cocatalyst on the right crystal facet of a photocatalyst, that is, reduction cocatalyst on the {010} facets and oxidation cocatalyst on the {110} facets, has been widely reported to enhance the photocatalytic activities of BiVO_4_.^[^ ^47^ ^]^ It is noted that majority of the subsequent studies of photocatalytic H_2_O_2_ production from BiVO_4_ have focused on the synergistic effects of crystal facet engineering and cocatalyst loading. Dual‐faceted BiVO_4_ with well‐defined {010} and {110} surfaces are adopted to leverage their inherent charge separation properties and enhance overall photocatalytic activity.

**Figure 5:**
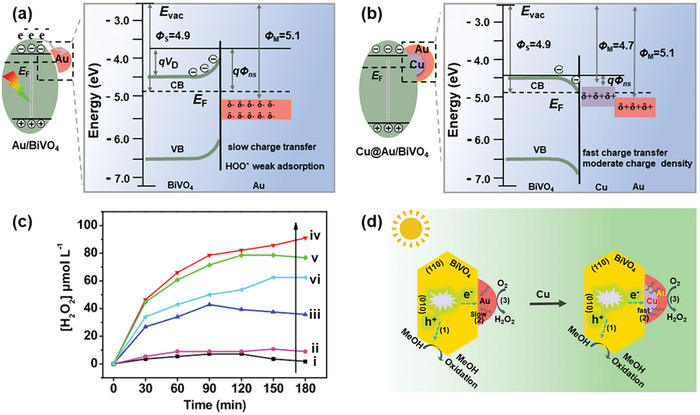
Utilization of core–shell Cu@Au as the metal cocatalyst for photocatalytic H_2_O_2_ production on BiVO_4_. Schematic diagrams of the band bending in BiVO_4_ and the charge density at the interface between a) BiVO_4_ and Au and b) BiVO_4_ and Cu@Au core–shell. Evac, *E*_F_, *Ф*, and *δ* represent the vacuum level, fermi level, work function, and charge density, respectively. c) Photocatalytic H_2_O_2_ production rates of various BiVO_4_‐based photocatalysts: i) bare BiVO_4_, ii) Cu/BiVO_4_, iii) Cu@Au/BiVO_4_ (Cu:Au molar ratio = 1:0.2), iv) Cu@Au/BiVO_4_ (1:1), v) Cu@Au/BiVO_4_ (1:3), and vi) Au/BiVO_4_ under visible‐light irradiation. d) Schematic illustration of the reaction mechanism for photocatalytic H_2_O_2_ production on Cu@Au/BiVO_4_. The process involves 1) spatial separation of charge carriers, 2) interfacial electron transfer, and 3) catalytic reaction on Au surface, respectively. Adapted with permission.^[^ ^28^ ^]^ Copyright 2021, American Chemical Society.

Although Au is a widely recognized metal cocatalyst, Wang et al. identified two main limitations that affect the performance of Au‐modified BiVO_4_ (illustrated in **Figure** **5**a): i) inefficient transfer of photogenerated electrons associated with built‐in potential (qV_D_) arising from upward band bending of BiVO_4,_ and ii) reduced catalytic activity for the two‐electron O_2_ reduction to H_2_O_2_.^[^ ^28^ ^]^ This reduction in activity is attributed to an increased negative density on Au upon its contact with BiVO_4_, leading to weaker adsorption of O_2_ and HOO* intermediates on the metal surface. To circumvent these limitations, they demonstrated the use of nanostructured Cu@Au core–shell particles as a more efficient metal cocatalyst compared to single‐metal cocatalysts (i.e., Au or Cu) for photocatalytic H_2_O_2_ generation from the faceted BiVO_4_. Owing to the lower work function of Cu (Ф_Cu_ = 4.7 eV) compared to Au (Ф_Cu_ = 5.1 eV), an ohmic contact can be formed between Cu and BiVO_4_, facilitating efficient electron transfer. This catalyst design reduces negative charge accumulation on Au, resulting in positive charge accumulation (Figure 5b), which was suggested to be beneficial for generating stronger adsorption of O_2_ and HOO* on the Au surface. Experimentally, Cu@Au/BiVO_4_ with the Cu@Au cocatalyst selectively formed on the {010} facets of BiVO_4_ and an optimized Cu:Au molar ratio of 1:1 showed the highest H_2_O_2_ production (91.1 µM under 3 h visible‐light irradiation in an aqueous methanol solution; Figure 5c). The superior performance of Cu@Au/BiVO_4_ was postulated to follow the reaction mechanism depicted in Figure 5d. After photoexcitation, the photogenerated electrons and holes in BiVO_4_ spatially separate to the {010} and {110} facets, respectively. The ohmic contact between Cu and BiVO_4_ on the {010} facets enables rapid electron transfer from BiVO_4_ to the Cu nanocore and subsequently to the catalytically active Au site, initiating the two‐electron reduction of O_2_.

**Figure 6:**
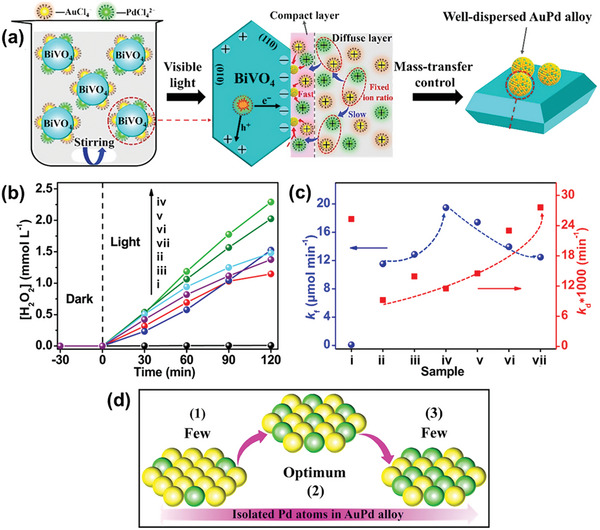
Utilization of AuPd alloy as the metal cocatalyst for photocatalytic H_2_O_2_ production on BiVO_4_. a) Schematic illustration of the formation process for uniformly dispersed AuPd alloy nanoparticles selectively on the {010} facet of BiVO_4_ via photodeposition. b) Time profile of photocatalytic H_2_O_2_ production in citrate buffer solution under visible‐light irradiation (λ = 420 nm). c) Kinetic parameters (*k*_f_ and *k*_d_) for H_2_O_2_ production on i) bare BiVO_4_, ii) Au‐BiVO_4_, iii) AuPd‐BiVO_4_ (Au:Pd mass ratio = 49:1), iv) AuPd‐BiVO_4_ (19:1), v) AuPd‐BiVO_4_ (9:1), vi) AuPd‐BiVO_4_ (2:1), and vii) Pd‐BiVO_4_. d) Schematic diagram illustrating the effect of increasing the Pd to Au mass ratio (from left to right) on the availability of isolated Pd atoms on the surfaces of the resulting AuPd alloys. Reproduced with permission.^[^ ^29^ ^]^ Copyright 2022, Elsevier.

Alloying Au with Pd presents an alternative strategy to surpass the limitations of single metal cocatalysts s and achieve enhanced photocatalytic performance. This method addresses the distinctive shortcomings of each metal cocatalyst: Au suffers from weak O_2_ binding, while Pd tends to favor the four‐electron reduction of O_2_ to H_2_O over the two‐electron reduction of O_2_ to H_2_O_2_.^[^ ^48^ ^]^ Using a photodeposition technique, Shi et al. successfully fabricated an AuPd alloy nanocatalyst with uniform atom dispersion, primarily supported on the {010} facet of BiVO_4_, as illustrated in **Figure** **6**a.^[^ ^29^ ^]^ While modification of the dual‐faceted BiVO_4_ with either Au or Pd improved the photocatalytic H_2_O_2_ performance compared to the bare material, incorporation of AuPd alloy nanocatalysts led to a more significant enhancement (Figure 6b). The AuPd‐BiVO_4_ catalyst with an optimized Au:Pd mass ratio of 19:1 achieved the highest H_2_O_2_ generation of 2.29 mM after 2 h of visible light irradiation in citrate buffer solution. Analysis of rate constants for H_2_O_2_ formation (*k*_f_) and decomposition (*k*_d_) revealed that as Pd content in the AuPd alloy increased, the *k*_d_ increased, while the *k*_f_ displays a volcanic trend, peaking at an Au:Pd mass ratio of 19:1 (Figure 6c). This mass ratio, leading to the best performing AuPd alloy supported on BiVO_4_, is attributed to the uniform dispersion of Au and Pd atoms. Further increasing the Au:Pd mass ratio beyond its optimal ratio leads to the formation of Pd atom clusters (Figure 6d), which are detrimental to the photocatalytic H_2_O_2_ reaction.

The discussion thus far has identified noble metal such as Au and Pd as promising cocatalysts for photocatalytic H_2_O_2_ production. Notably, Shi et al. demonstrated the potential of nickel sulfide (NiS) as an effective, low‐cost, non‐precious metal alternative cocatalyst to enhance the photocatalytic H_2_O_2_ production of BiVO_4_.^[^ ^30^ ^]^ The NiS cocatalyst was selectively deposited on the {010} facet of BiVO_4_, which is the electron‐rich, using a sulfur‐mediated photodeposition method. In this process, Ni^2+^ ions are electrostatically adsorbed onto the electron‐rich {010} facet of BiVO_4_ under visible‐light illumination, followed by in situ NiS formation through the reaction between the enriched Ni^2+^ and surrounding S molecules. The optimal NiS/BiVO_4_ photocatalyst (10 wt% NiS) achieved an H_2_O_2_ production concentration of 975 µM, which is 87 times higher than that of unmodified BiVO_4_ (11.25 µM), with an AQY of 4.8% under 420 nm irradiation. The NiS cocatalyst was found to enhance O_2_ adsorption on the BiVO_4_ surface. The selective loading on the {010} facet facilitates the effective capture of photogenerated electrons, thereby promoting the reduction of adsorbed O_2_ molecules to H_2_O_2_.

**Figure 7:**
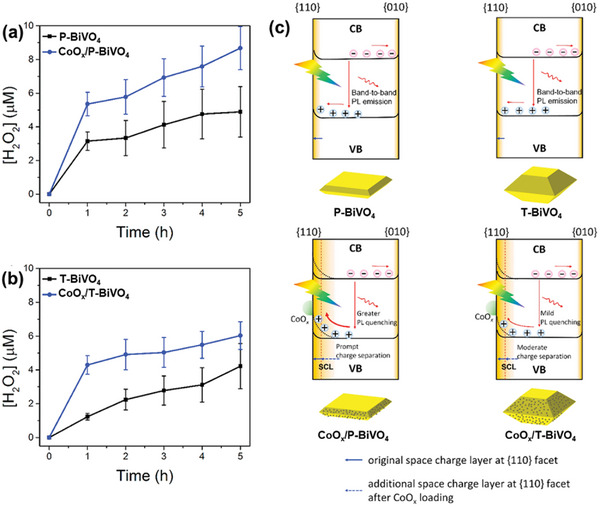
Enhanced photocatalytic H_2_O_2_ production by BiVO_4_ coupled with CoO*_x_* as the WOR cocatalyst. a,b) Time profiles of H_2_O_2_ production from a) P‐BiVO_4_ and P‐BiVO_4_/CoO*_x_* and b) T‐BiVO_4_ and T‐BiVO_4_/CoO*_x_* under visible‐light irradiation (λ > 420 nm) in the presence of methanol as the hole scavenger. CoO*_x_* was deposited on each BiVO_4_ support via photodeposition. While both BiVO_4_ types exhibited increased H_2_O_2_ production with CoO*_x_*, the enhancement degree is more pronounced for P‐BiVO_4_. c) Schematic diagrams comparing the space charge layer thickness in P‐BiVO_4_ and T‐BiVO_4_ before and after CoO*_x_* deposition. Adapted with permission.^[^ ^31^ ^]^ Copyright 2022, Elsevier.

Apart from loading ORR cocatalyst, deposition of WOR cocatalyst can also improve the photocatalytic H_2_O_2_ production performance of BiVO_4_. Xie et al. showed that the H_2_O_2_ yield of dual‐faceted BiVO_4_ can be enhanced by selectively loading CoO*_x_* (a known cocatalyst for WOR) onto the {110} reduction functional facet.^[^ ^31^ ^]^ Interestingly, the degree of enhancement was uncovered to be dependent on the relative exposure ratio of {010}/{110} facets in BiVO_4_. P‐BiVO_4_, with a {010} dominant facet, exhibited a 79.2% increase, while {110}‐dominant T‐BiVO_4_ showed a 42.9% increase (**Figure** **7**a,b). The superior performance of CoO*_x_*/P‐BiVO_4_ was ascribed to the synergistic effects of a larger built‐in electric field in P‐BiVO_4_ compared to T‐BiVO_4_ and a greater degree of band bending at the interface upon heterojunction formation with CoO*_x_*. These factors result in a larger space charge layer at the {110} facets of CoO*_x_*/P‐BiVO_4_ compared to CoO*_x_*/T‐BiVO_4_ (Figure 7c), ultimately leading to enhanced charge separation and photoactivity in CoO*_x_*/P‐BiVO_4_.

**Figure 8:**
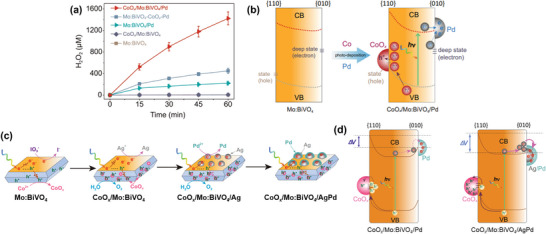
Selective cocatalyst deposition on BiVO_4_ for enhanced H_2_O_2_ production. a) Time profiles of photocatalytic H_2_O_2_ generation under simulated solar light irradiation for various photocatalysts: CoO*_x_*/Mo:BiVO_4_/Pd (selective deposition), CoO*_x_*/Mo:BiVO_4_, Mo:BiVO_4_/Pd, and Mo:BiVO_4_‐CoO*_x_*‐Pd (random deposition). b) Schematic diagram illustrating the effects of CoO*_x_* and Pd coloading on the energetics of exposed {110} and {010} facets on Mo:BiVO_4_. Reproduced with permission.^[^ ^32^ ^]^ Copyright 2022, Spring Nature. c) Schematic illustration of stepwise photodeposition of Co, Ag, and Pd on Mo:BiVO_4_, resulting in the selective formation of CoO*_x_* and core–shell AgPd cocatalysts on the {110} and {010} facets of Mo:BiVO_4_, respectively. d) Schematic illustration of enhanced charge separation due to surface energetics tuning by the formation of core–shell AgPd cocatalyst compared to Pd. Adapted with permission.^[^ ^33^ ^]^ Copyright 2022, Spring Nature.

Liu et al. recently reported a breakthrough in photocatalytic H_2_O_2_ production, achieving the highest efficiency among inorganic photocatalysts. By strategically loading dual cocatalysts onto specific facets of Mo‐doped BiVO_4_ (Mo:BiVO_4_), they greatly enhanced the material's performance.^[^ ^32^, ^33^ ^]^ Molybdenum was doped into the BiVO_4_ crystal structure by replacing the V sites, thereby increasing the bulk conductivity of the material. In their initial study, selective deposition of Pd cocatalyst on the {010} facets of Mo:BiVO_4_ (Mo:BiVO_4_/Pd) significantly enhanced the H_2_O_2_ production yield (53.7‐fold compared to bare Mo:BiVO_4_; 4.1 µM for bare Mo:BiVO_4_ and 220.1 µM for Mo:BiVO_4_/Pd after 1 h of simulated light irradiation). The subsequent loading of CoO*_x_* cocatalyst onto the {110} facets resulted in a remarkable 347.6‐fold enhancement (**Figure** **8**a). Specifically, CoO*_x_*/Mo:BiVO_4_/Pd achieved a H_2_O_2_ production rate of 1425 µM h^−1^, surpassing the photocatalytic H_2_O_2_ production performance of previously reported inorganic photocatalysts by an order of magnitude, and without the presence of any sacrificial reagent.^[^ ^32^ ^]^ The photocatalytic H_2_O_2_ production reaction was proved to proceed mainly through the two‐electron ORR. Beyond the improved surface kinetics for O_2_ evolution and H_2_O_2_ selectivity facilitated by CoO*_x_* and Pd as WOR and ORR cocatalysts, respectively, the coloading of these cocatalysts on selective BiVO_4_ facets was experimentally probed using transient absorption spectroscopy (TAS) to enhance charge separation within the material, as illustrated in Figure 8b. The presence of cocatalysts not only facilitates the accumulation of photogenerated holes in Mo:BiVO_4_ and promotes the transfer of free/shallowly trapped photogenerated electrons to Pd, but also activates and transfer the deeply trapped electrons to Pd for subsequent surface reactions.

In a subsequent study, Liu et al. further improved the performance by replacing Pd with an AgPd core/shell structure cocatalyst, which was obtained by stepwise photoreduction of Ag^+^ and PdCl_4_^2−^ (Figure 8c).^[^ ^33^ ^]^ The Ag core was reported to lower the Schottky barrier at the BiVO_4_/cocatalyst interface, as it has a lower work function (4.3 eV) compared to Pd (5.6 eV), while retaining the Pd shell to promote two‐electron ORR to H_2_O_2_. Such a reduction in the Schottky barrier at the {010} facets through the construction of a BiVO_4_/Ag junction enhances electron migration and charge separation, as evidenced by TAS measurement (Figure 8d). The optimized CoO*_x_*/Mo:BiVO_4_/AgPd composite achieved a record‐breaking H_2_O_2_ production rate of 9.7 mM h^−1^ with an AQY of 13.1% at 420 nm, setting a new benchmark for inorganic semiconductors.

Rare earth elements such as yttrium (Y) and gadolinium (Gd) have also been investigated as effective dopants for BiVO_4_ in photocatalytic H_2_O_2_ production. Due to the similar ionic radii of Y^3+^ (0.90 Å), Gd (1.05 Å), and Bi^3+^ (1.03 Å), Y^3+^ or Gd^3+^ can substitute for Bi^3+^ in the BiVO_4_ lattice.^[^ ^34^, ^35^ ^]^ Dai et al. demonstrated the feasibility of doping BiVO_4_ with Y^3+^ using a hydrothermal method, resulting in a fourfold increase in the H_2_O_2_ production rate for the optimized Pd/Y‐doped BiVO_4_ (114 µmol g^−1^ h^−1^) compared to undoped Pd/BiVO_4_ (26 µmol g^−1^ h^−1^) under simulated sunlight (AM 1.5) irradiation.^[^ ^34^ ^]^ DFT calculations suggested that Y doping enhances O_2_ adsorption on the BiVO_4_ surface, while experimental results revealed that it induces monoclinic/tetragonal heterojunction formation, which further promotes charge separation and enhances photocatalytic activity for H_2_O_2_ production. On the other hand, Gd‐doped BiVO_4_ was also successfully fabricated by Li et al. via hydrothermal approach.^[^ ^35^ ^]^ Au was selectively deposited on the {010} facets of the resulting Gd‐doped BiVO_4_ as a cocatalyst. The optimized Au/Gd‐doped BiVO_4_ demonstrated significantly enhanced photocatalytic performance, producing 2.07 mM of H_2_O_2_ with an AQY of 7.44% within 2 h under visible‐light irradiation, which is a 1.39‐fold increase in activity compared to the undoped Au/BiVO_4_. Gd doping was found to inhibit H_2_O_2_ decomposition and lower the Fermi energy level of BiVO_4_ to better match that of Au. This adjustment reduces the Schottky barrier, facilitating faster charge transfer and improving H_2_O_2_ production.

**Figure 9:**
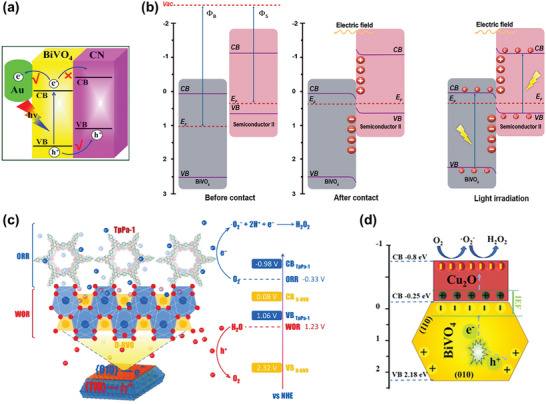
Heterojunction formation for enhanced BiVO_4_ photocatalytic H_2_O_2_ production. Charge transfer mechanisms for a) C_3_N_4_/BiVO_4_/Au Type‐II heterojunction and b) S‐scheme heterojunctions achieved using NiCo_2_O_4_/BiVO_4_, Cu_2_O/BiVO_4_, PTA/BiVO_4_, TpPa‐1‐ COF/BiVO_4_ composites. Reproduced with permission.^[^ ^36^ ^]^ Copyright 2021, Elsevier. c,d) Schematic illustration of the proposed reaction pathways for H_2_O_2_ production over c) TpPa‐1‐ COF/BiVO_4_ and d) Cu_2_O/BiVO_4_. The heterojunctions are formed primarily on the {010} facets of BiVO_4_ to facilitate directional charge transfer. Reproduced with permission.^[^ ^40^ ^]^ Copyright 2024, Elsevier. Reproduced with permission.^[^ ^39^ ^]^ Copyright 2024, Elsevier.

The formation of a heterojunction with another photocatalyst exhibiting appropriate band alignment presents an alternative strategy to mitigate the intrinsic limitation of the high charge recombination rate in bare BiVO_4_. Shi et al. showcased the formation of a Type II heterojunction by coupling BiVO_4_ with C_3_N_4_, where photogenerated electrons migrate from C_3_N_4_ to BiVO_4_, and holes move in the opposite direction (**Figure** **9**a).^[^ ^36^ ^]^ Strategically, presynthesized ultrathin C_3_N_4_ was electrostatically attracted primarily on the hole‐rich {110} facets of BiVO_4_ to promote efficient transfer of photogenerated holes, whereas Au nanoparticles were photodeposited onto the electron‐rich {010} facets to facilitate the two‐electron ORR necessary for H_2_O_2_ generation. The resulting C_3_N_4_/BiVO_4_/Au composite exhibited superior H_2_O_2_ production, achieving 1351.78 µM in 2h, which is 2.65‐ and 150.5‐fold higher than that of Au/BiVO_4_ and BiVO_4_, respectively. The incorporation of C_3_N_4_ functions as a photocatalytic H_2_O_2_ decomposition suppresser due to its weaker valence band oxidation potential, which limits its capacity to oxidize H_2_O_2_ to O_2_.

The step‐scheme (S‐scheme) heterojunction has also been identified as an effective method to enhance the photocatalytic performance of BiVO_4_ for H_2_O_2_ production. In an S‐scheme heterojunction, BiVO_4_ is typically paired with a photocatalyst that has a higher CB to compensate for BiVO_4_’s relatively weak reducing potential. When these materials come into contact, electrons spontaneously transfer from the photocatalyst with the higher CB to BiVO_4_ until equilibrium is reached at the Fermi level. This process leads to band bending and charge redistribution, creating an internal electric field (IEF) at the interface. Under irradiation, this IEF drives photogenerated electrons from BiVO_4_ to recombine with photogenerated holes from the other photocatalyst, resulting in the retention of electrons in the CB of the latter and holes in the VB of BiVO_4_ (Figure 9b). This S‐scheme heterojunction effectively promotes interfacial charge transfer, enhances charge separation, and preserves energetic charge carriers. BiVO_4_ has been successfully integrated into S‐scheme heterojunctions with NiCo_2_O_4_,^[^ ^37^ ^]^ Cu_2_O,^[^ ^39^ ^]^ perylenetetracarboxylic acid (PTA),^[^ ^38^ ^]^ and TpPa‐1‐covalent organic framework (COF)^[^ ^40^ ^]^ to improve photocatalytic H_2_O_2_ generation. Due to the higher reducing potential of the electrons in the additional photocatalyst that serves as the reduction site, H_2_O_2_ generation in these S‐scheme heterojunctions occurs via an indirect stepwise one‐electron transfer reaction (O_2_ + e^−^ → ^•^O_2_^−^ + e^−^ + 2H^+^ → H_2_O_2_) during the ORR, while H_2_O is oxidized to O_2_ in the oxidation half‐reaction on BiVO_4_. Of note, the TpPa‐1‐COF/BiVO_4_ and Cu_2_O/BiVO_4_ S‐scheme heterojunctions underscores the importance of crystal facet engineering in the rational design of highly effective hybrid photocatalysts.^[^ ^39^, ^40^ ^]^ The COF and Cu_2_O were intentionally grown primarily on the electron‐rich {010} facets of BiVO_4_ (as shown in Figure 9c,d, respectively) to facilitate directional interfacial charge transfer. These composites exhibited superior photoactivity, which was attributed to enhanced charge separation compared to their counterparts formed randomly.

**Figure 10:**
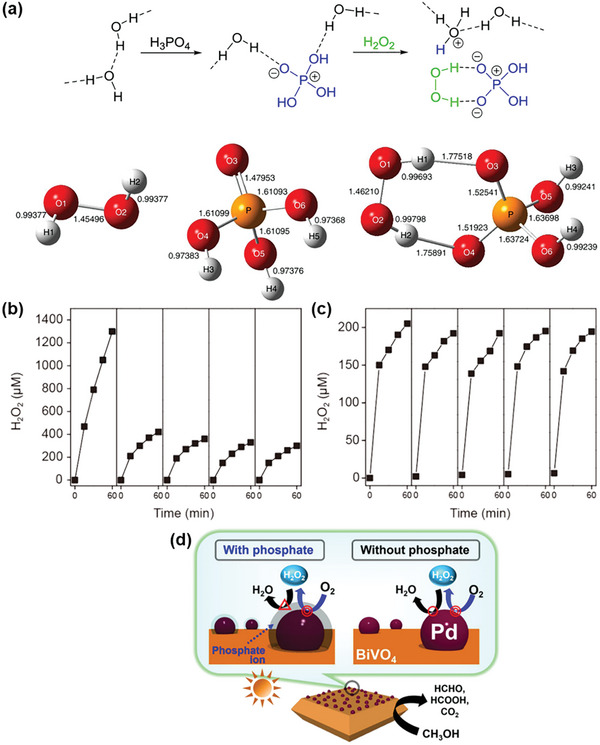
Summary of phosphate (PO_4_^3−^) as H_2_O_2_ stabilizer. a) Schematic representation of the H‐bonding interaction between water, H_2_PO_4_^−^, and H_2_O_2_ molecules, along with optimized structures and bond lengths calculated by density functional theory (DFT). Reproduced with permission.^[^ ^50^ ^]^ Copyright 2020, Springer Nature. Time profiles of photocatalytic H_2_O_2_ production on CoO*_x_*/Mo:BiVO_4_/Pd catalysts in b) KPi aqueous solution and c) pure water for five repeated cycles under solar simulator light irradiation. Reproduced with permission.^[^ ^32^ ^]^ Copyright 2022, Spring Nature. d) Schematic illustration of the H_2_O_2_ decomposition inhibition mechanism by phosphate‐coated Pd supported on BiVO_4_, obtained via the photodeposition method using a phosphate buffer solution. Reproduced with permission.^[^ ^27^ ^]^ Copyright 2020, Elsevier.

Another strategy to promote H_2_O_2_ generation involves the inclusion of phosphate (PO_4_^3−^) into the reaction system as an H_2_O_2_ stabilizer. Phosphoric acid (H_3_PO_4_) is generally employed to stabilize commercially available H_2_O_2_ and inhibit its decomposition.^[^ ^49^ ^]^ The interaction between H_2_O_2_ and H_3_PO_4_ was examined by Shiraishi et al. using Raman spectroscopy and ab initio calculations, as illustrated in **Figure** **10**a.^[^ ^50^ ^]^ Their findings suggest that H_3_PO_4_ forms a stabilized H_2_O_2_‐H_2_PO_4_^−^ bidentate complex through hydrogen bonding. The efficacy of PO_4_^3−^ as a H_2_O_2_ stabilizer in the photocatalytic H_2_O_2_ generation system was demonstrated by Liu et al., in which they reported a lower cumulative H_2_O_2_ yield in pure water compared to a phosphate buffer solution using CoO*_x_*/Mo:BiVO_4_/Pd (Figure 10b,c).^[^ ^32^ ^]^ However, PO_4_^3−^ was also found to react with the CoO*_x_* cocatalyst, transforming it into Co‐Pi and leading to a drastic performance decline during cyclic photocatalytic H_2_O_2_ generation reactions with phosphate solution (Figure 10b). On the other hand, Fuku et al. showcased improved BiVO_4_ photoactivity with partial phosphate (PO_4_^3−^) coating onto the Pd nanoparticles, achieving an optimal H_2_O_2_ yield of *≈*600 µM.^[^ ^27^ ^]^ This represents a marked increase from the 340 µM yield obtained with uncoated Pd‐loaded BiVO_4_ under identical experimental conditions (2 h irradiation, 0.1 wt% Pd). The superior H_2_O_2_ production performance of the PO_4_^3−^‐coated Pd‐loaded BiVO_4_ was attributed to its ability to effectively inhibit H_2_O_2_ degradation, as depicted in Figure 10d.

## 4 Photoelectrochemical (PEC) System

**Table** **3** outlines the performance of BiVO_4_‐based photoelectrodes reported to date for PEC H_2_O_2_ generation. These photoelectrodes, typically employing n‐type BiVO_4_ as the light absorber, function primarily as photoanodes, catalyzing H_2_O_2_ production through two‐electron water oxidation. Notably, there have been significant advancements in the material's PEC H_2_O_2_ generation performance in less than a decade, with a FE exceeding 85% attained at applied biases lower than the theoretical electrolysis voltage (+1.77 V versus RHE).^[^ ^43a,c^ ^]^ Moreover, BiVO_4_‐based materials have demonstrated the potential to generate H_2_O_2_ concurrently on both the photoanode and cathode without an external bias. These advancements have been made possible through various strategies, including material engineering and reaction conditions optimization, which will be elaborated upon in this section. To establish a thorough understanding of photoelectrode development, we will first provide an overview of the experimental characterization methods used to assess the performance of PEC systems for H_2_O_2_ generation.

**Table 3:** Summary of PEC H_2_O_2_ production using BiVO_4_‐based materials as photoanodes.

Photoanode	Cathode	Experimental Conditions					Reaction Type	H_2_O_2_ Production Rate [µmol min^−1^ cm^−2^]	FE(H_2_O_2_) [%]	ABPE [%]^a)^	Ref
		Light	Electrolyte	Temperature Control [°C]	Membrane	Applied Bias [V versus RHE]					
WO_3_/BiVO_4_	Pt	λ > 420 nm	2M KHCO_3_ (pH 7.7), CO_2_ bubbling	Ice bath (< 5°C)	Yes	1.5	Anodic Oxidation	0.18^b)^	54	2.2	[22]
WO_3_/BiVO_4_	Au	AM 1.5G	2M KHCO_3_ (pH 7.8), O_2_ and CO_2_ bubbling	Ice bath	Both	Bias‐free	Dual‐sided	1.12^b)^	Anode: *≈*50 Cathode: *≈*90	–	[41b]
WO_3_/BiVO_4_/Al_2_O_3_ (MOD)	Pt	λ > 420 nm	2M KHCO_3_ (pH 7.7)	Ice bath (< 5°C)	Yes	1.5	Anodic Oxidation	0.45^b)^	79	1.5	[41a]
WO_3_/BiVO_4_/Al_2_O_3_ (CVD)	Pt	AM 1.5G	2M KHCO_3_ (pH 7.0–7.8) CO_2_ bubbling	Ice bath	Yes	0.8	Anodic Oxidation	0.86^b)^	80	2.57	[41c]
	Biomass‐derived carbon (WSoy/GnP‐CP)	AM 1.5G	Anode: 2M KHCO_3_ (pH 7.9), CO_2_ bubbling Cathode: 2M KHCO_3_ (pH 8.6), O_2_ bubbling	Ice bath (0–5°C)	Yes	Bias‐free	Dual‐sided	0.58^b)^	Anode: 60 Cathode: 44	–	[41c]
SnO_2‐_ *_x_* /BiVO_4_	Pt	AM 1.5G	1M NaHCO_3_ (pH 8.3)	–	Yes	1.23	Anodic Oxidation	0.825	86	–	[43a]
ZnO/BiVO_4_	Pt	AM 1.5G	2M KHCO_3_ (pH 8.3)	–	Yes	1.4	Anodic Oxidation	0.222	*≈*39.7	–	[44a]
BiVO_4_‐Air/V	Pt	AM 1.5G	1M NaHCO_3_ (pH 8.3)	–	Yes	1.23	Anodic Oxidation	–	*≈*58.4	–	[51]
O‐BiVO_4_	Pt	AM 1.5G	2M KHCO_3_ (pH 8.3)	–	Yes	1.6	Anodic Oxidation	0.04	50.2	–	[52]
N‐O_vac_‐BiVO_4_	Pt	AM 1.5G	1M NaHCO_3_ (pH 8.3)	–	Yes	1.6	Anodic Oxidation	0.15^b)^	81.2	1.1	[43b]
PTFE/BiVO_4_	Pt	AM 1.5G	1M NaHCO_3_ (pH 8.3)	–	Yes	1.23	Anodic Oxidation	0.05^b)^	85	–	[43c]
Mo‐doped BiVO_4_	Pt	AM 1.5G	1M NaHCO_3_ (pH ≈ 7.8)	–	Yes	1.0	Anodic Oxidation	0.13	35	–	[53]
	AQ‐modified Carbon	AM 1.5G	1M NaHCO_3_ (pH ≈ 7.8)	–	Yes	1.0	Dual‐sided	0.45	Anode: *≈*40 Cathode: *≈*100	–	
		AM 1.5G	1M NaHCO_3_ (pH ≈ 7.8)	–	Yes	Bias‐free	Dual‐sided	0.11	Anode: *≈*25 Cathode: *≈*100	–	
Phosphate‐treated Mo‐doped BiVO_4_	AQ‐modified Carbon	AM 1.5G	1M NaHCO_3_ (pH ≈ 7.8)	–	Yes	1.0	Dual‐sided	0.66	Anode: *≈*48 Cathode: *≈*100	–	[53]
		AM 1.5G	1M NaHCO_3_ (pH ≈ 7.8)	–	Yes	Bias‐free	Dual‐sided	0.16	Anode: *≈*43 Cathode: *≈*100	–	
WO_3_(NN/NH)/Mo‐doped BiVO_4_	Pt	AM 1.5G	2M KHCO_3_, CO_2_ bubbling	Ice bath	Yes	0.8	Anodic Oxidation	1.15	–	–	[54]
Co^2+^ and PO_4_^3−^ ions modified Mo‐doped BiVO_4_	Pt	AM 1.5G	Anode: 2M KHCO_3_ (pH ≈ 8.3), CO_2_ bubbling Cathode: 0.2M Na_2_SO_4_ (pH ≈ 7)	Ice bath (5°C)	Yes	1.7	Anodic Oxidation	0.23	26	–	[41d]
Gd‐doped BiVO_4_	Carbon	AM 1.5G	2M KHCO_3_ (pH ≈ 8.3)	–	No	2.6	Anodic Oxidation	1.1	*≈*99.5	–	[55]
(010)‐dominant BiVO_4_	Pt	AM 1.5G	1M NaHCO_3_ (pH 8.3)	–	Yes	0.93	Anodic Oxidation	–	*≈*70	0.94	[43e]
BiVO_4_	Graphite	λ = 420 nm	Carbon quantum dots aqueous solution	Ice bath (< 5°C)	No	1.23	Anodic Oxidation	0.33	93.5	–	[43d]
BiVO_4_	Carbon	AM 1.5G	Anode: 2M KHCO_3_ (pH 8.3), O_2_ bubbling Cathode: 1M Na_2_SO_4_ (pH 8.3), O_2_ bubbling	Ice bath	Yes	1.5^c)^	Dual‐sided	2.42	–	–	[56]
			Tap water, O_2_ bubbling	Ice bath	Yes	1.5^c)^	Dual‐sided	0.342	–	–	
			Distilled water, O_2_ bubbling	Ice bath	Yes	1.5^c)^	Dual‐sided	0.225	–	–	
			Anode: 2M KHCO_3_ (pH ≈ 8.3), O_2_ bubbling Cathode: 1M Na_2_SO_4_ (pH ≈ 8.3), O_2_ bubbling	Ice bath	Yes	Bias‐free	Dual‐sided	0.48	Anode: 53 Cathode: 92	–	

^a)^ Calculated at the applied bias with optimal operating conditions;

^b)^ Estimated based on the reported amount of H_2_O_2_ produced, measurement time, irradiated area of photoanode, and/or geometric area of cathode;

^c)^ Applied bias of anode versus Cathode.

### 4.1 Evaluation of PEC Performance for H_2_O_2_ Generation

The performance of a photocatalyst for PEC H_2_O_2_ generation can be quantified and analyzed through three key parameters: activity, selectivity, and stability. Electrocatalytic activity is typically determined using either cyclic voltammetry (CV) or linear sweep voltammetry (LSV) to obtain the current–voltage (*J*–*V*) curve under both dark and illuminated conditions. For a detailed exploration of proper experimental protocols for voltammetric measurements and assessment, a comprehensive review paper by Shi et al. is highly recommended.^[^ ^57^ ^]^ A one‐ or two‐compartment PEC configuration can be employed with a BiVO_4_‐based photoanode, a counter electrode such as a Pt wire, and a reference electrode such as Ag/AgCl. However, a two‐compartment configuration equipped with an ion‐exchange membrane (i.e., Nafion) is generally preferred. This configuration minimizes the cathodic degradation of H_2_O_2_ generated at the photoanode during the WOR.^[^ ^18b^ ^]^ The electrolyte is commonly cooled in an ice bath (below 5 °C) to avoid further thermal decomposition of H_2_O_2_.

The onset potential, overpotential, and photocurrent density serve as crucial parameters for comparing the electrocatalytic activity of photoelectrodes fabricated with various photocatalysts. Due to the thermodynamically unfavorable nature of two‐electron WOR for H_2_O_2_, the *J*–*V* onset might be attributed to the evolution of O_2_ via four‐electron WOR. To ensure precise measurement of the onset potential for H_2_O_2_ generation, Shi et al. suggested defining it as the voltage at which the H_2_O_2_ concentration reaches 1 ppm.^[^ ^24b^ ^]^ Conversely, the onset potential can also be evaluated as the voltage where the current density toward H_2_O_2_ (*J*(H_2_O_2_)) is 1 mA cm^−2^. *J*(H_2_O_2_) is determined by multiplying the overall current density with the voltage‐dependent Faraday efficiency (FE(H_2_O_2_)).^[^ ^57^ ^]^ The overpotential for H_2_O_2_ generation (ƞ(H_2_O_2_)) signifies the additional voltage required to drive this anodic reaction beyond the thermodynamic limit (1.77 V versus RHE), and can then be calculated using the following equation:

(16) $$\eta \left(H_{2}O_{2}\right)=\mathrm{on}\;\mathrm{set}\;\mathrm{potential}-1.77$$

Material performance can also be evaluated by analyzing the current densities generated at a constant voltage. While some levels of H_2_O_2_ generation can occur electrochemically in the dark, light irradiation of the photoanode readily lowers the onset potential, improves the current density, and increases FE(H_2_O_2_).

The detection and quantification of generated H_2_O_2_, typically in the liquid phase, employ methodologies similar to those documented for PS system, are summarized in Table 2. However, disparities in experimental setups, such as the photoelectrode's irradiation area and measurement time, necessitates careful consideration when comparing H_2_O_2_ production performance across studies. For a meaningful comparison, the reported amount of H_2_O_2_ generated should be normalized by both the irradiation area and reaction duration (i.e., µmol min^−1^ cm^−2^). The presence of the competing O_2_ evolution reaction, where generated O_2_ can exist in either the gaseous or dissolved phase, can be verified and quantified using gas chromatography equipped with a thermal conductivity (TCD) detector or an O_2_ sensor.

Faraday efficiency (FE) serves as a key performance metric to evaluate the selectivity of a PEC system toward a desired product. It measures the ratio between the actual amount of product generated and the theoretically amount expected based on the measured current and the assumption of perfect efficiency. The FE for H_2_O_2_, O_2_, and H_2_ production can be calculated using the following equation:^[^ ^57^ ^]^

(17) $$\begin{array}{ccc} \mathrm{FE}\left(\mathrm{Product}\right)\;\left(\%\right) & & =\frac{\mathrm{Amount}\;\mathrm{of}\;\mathrm{generated}\;\mathrm{product}}{\mathrm{Theoretical}\;\mathrm{amount}\;\mathrm{of}\;\mathrm{product}}\;\times 100\\ & & =\frac{N_{\mathrm{product}}}{\frac{\int_{0}^{t}I\left(t\right)dt}{nF}}\;\times 100 \end{array}$$

where *N*_product_ is the amount of generated product (i.e., H_2_O_2_, O_2_, or H_2_) in moles, *I*(*t*) is the current measured as a function of time under the applied bias, *t* is the reaction duration (s), *F* is the Faraday constant (96485 C mol^−1^), and *n* is the number of electrons required to create one molecule of the product (2 for H_2_O_2_ and H_2_ generation; 4 for O_2_ production). Since FE is dependent on the applied bias, it is typically determined at various voltages. At a constant bias, the sum of FE of all oxidation products at anode should ideally approach 100% to avoid overestimation, with an analogous principle applying to the cathode for reduction products.

In addition to FE, solar energy conversion efficiency, or half‐cell applied‐bias photon‐to‐current efficiency (ABPE), can also be used to assess the efficiency of a photoanode. For BiVO_4_‐based photoanode generating H_2_O_2_ and O_2_ as the oxidation products, ABPE can be determined using the following equation:

(18) $$\begin{array}{ccc} A\mathrm{BPE}\;\;\left(\%\right) & & =\left[\frac{J_{\mathrm{opt}}\times \left(1.77\;V-\;E_{\mathrm{opt}}\right)\times \mathrm{FE}\left(H_{2}O_{2}\right)}{P_{\mathrm{int}}}\right]\\ & & +\left[\frac{J_{\mathrm{opt}}\times \left(1.23\;V-\;E_{\mathrm{opt}}\right)\times \mathrm{FE}\left(O_{2}\right)}{P_{\mathrm{int}}}\right] \end{array}$$

where *J*_opt_ represents the photocurrent density at the applied bias with optimal operating conditions (*E*_opt_) and *P*_int_ denotes the intensity of the light source.

Chronoamperometry is the primary technique used to assess photoelectrode stability. This technique involves measuring the photocurrent density over an extended period under a constant applied bias. A stable photoelectrode exhibits minimal degradation, as evidenced by a steady photocurrent density maintained over several hours to days. However, there is currently no standardized metric for comparing photoanode stability for water oxidation.

### 4.2 Strategies for Enhanced PEC H_2_O_2_ Production

#### 4.2.1 Bias‐Assisted, Anodic H_2_O_2_ Generation

**Figure 11:**
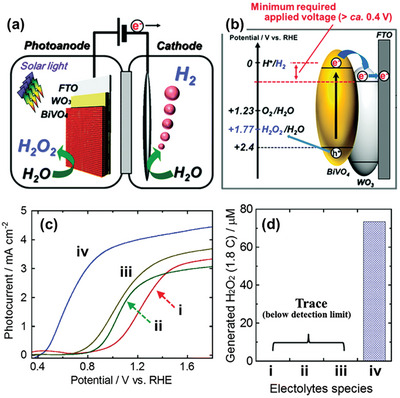
PEC system for simultaneous H_2_O_2_ and H_2_ production. a) Schematic diagram of the PEC system, consisting of a WO_3_/BiVO_4_ photoanode and a Pt cathode. b) Energy diagram illustrating the band positions of WO_3_, BiVO_4_, and the redox potentials of water oxidation and reduction reactions. c) Photocurrent–voltage (*J*–*V*) curves and d) H_2_O_2_ production yields of the PEC system in 0.5 M solutions of different electrolytes: i) K_2_SO_4_, ii) phosphate buffer, iii) H_3_BO_3_, and iv) KHCO_3_ after passing a fixed electric charge of 1.8 C (1 mA for 30 min) under 1 sun irradiation. Reproduced with permission.^[^ ^22^ ^]^ Copyright 2016, Royal Society of Chemistry.

Sayama et al. have been at the forefront of designing WO_3_/BiVO_4_ heterojunctions photoanodes for electrochemical and PEC H_2_O_2_ production applications,^[^ ^22^, ^24^, ^41^ ^]^ with Fuku and Sayama achieving a landmark demonstration of H_2_O_2_ generation through H_2_O oxidation in a PEC system. They utilized a WO_3_/BiVO_4_ photoanode alongside concurrent H_2_ evolution on a Pt cathode (as depicted in **Figure** **11**a), accomplishing this feat at a significantly lower applied bias (<1 V versus RHE) than the theoretical electrolysis voltage (+1.77 V versus RHE) under simulated solar light.^[^ ^22^ ^]^ The WO_3_/BiVO_4_ photoanode was designed by coating BiVO_4_ on a WO_3_ underlayer to facilitate the transfer of photogenerated electrons from BiVO_4_ to the FTO substrate (Figure 11b). Meanwhile, photogenerated holes on the BiVO_4_ surface oxidized H_2_O to H_2_O_2_. The choice of KHCO_3_ electrolyte proved to be a key factor for driving oxidative H_2_O_2_ generation on the WO_3_/BiVO_4_ photoanode. Compared to other electrolytes (i.e., K_2_SO_4_, phosphate buffer, and H_3_BO_3_), KHCO_3_ displayed the highest photocurrent generation across a wide applied bias range (Figure 11c), and H_2_O_2_ production was detected exclusively in the electrolyte (Figure 11d). Through optimization of the HCO_3_^−^ concentration at 2 M under CO_2_ bubbling, a yield of ≈2 mM of H_2_O_2_ was achieved under visible‐light irradiation (λ > 420 nm) and at 1.5 V versus RHE, despite a maximum FE(H_2_O_2_) of merely 54%. O_2_ was reported as the only oxidative byproduct at the photoanode. The beneficial role of the KHCO_3_ electrolyte was attributed to the formation of percarbonate intermediates, such as HCO_4_^−^ and C_2_O_6_^2−^, resulting from the oxidation of HCO_3_^−^ by the photogenerated holes in BiVO_4_. Subsequent hydrolysis of these percarbonate species by H_2_O led to the formation of H_2_O_2_ and HCO_3_^−^. High concentrations of HCO_3_^−^ were found to not only promote H_2_O_2_ formation, but also aid in inhibiting H_2_O_2_ degradation at the photoanode.^[^ ^22^, ^41^ ^]^

**Figure 12:**
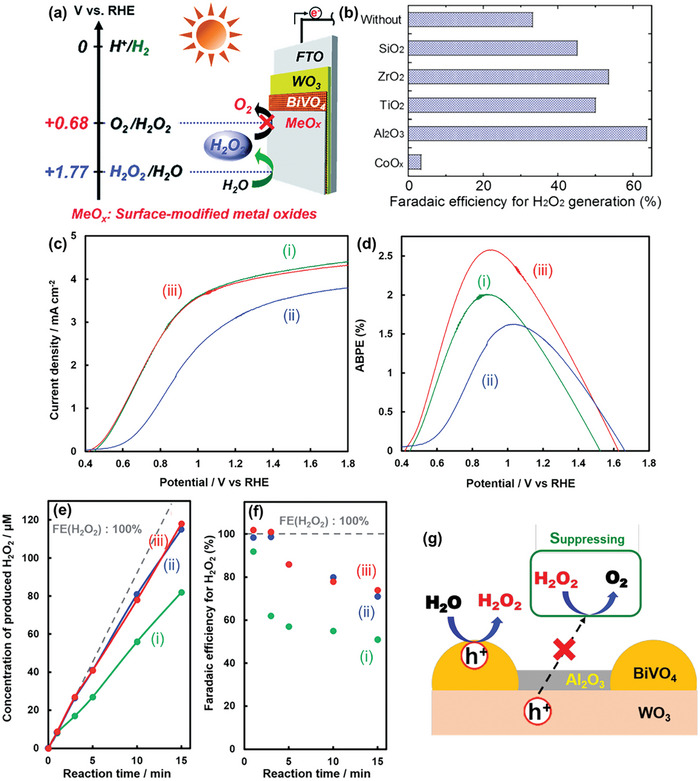
Effect of Al_2_O_3_ overlayer on WO_3_/BiVO_4_ photoanode for H_2_O_2_ production. a) Schematic diagram of PEC system for H_2_O_2_ generation from water oxidation on a metal‐oxide (MeO*_x_*)‐coated WO_3_/BiVO_4_ photoanode under solar light irradiation. b) Faradic efficiency (FE) for H_2_O_2_ generation from BiVO_4_/WO_3_ photoanodes modified with various MeO*_x_* using the MOD method. The measurements were conducted at an electric charge of 0.9 C (1 mA for 900 s) in a 0.5 M aqueous KHCO_3_ electrolyte at < 5 °C under simulated solar light. Reproduced with permission.^[^ ^41a^ ^]^ Copyright 2017, Royal Society of Chemistry. c) Photocurrent density and d) applied‐bias photon‐to‐current efficiency (ABPE) values for H_2_O_2_, O_2_, and H_2_ production in a PEC system using photoanodes of i) WO_3_/BiVO_4_, ii) WO_3_/BiVO_4_/Al_2_O_3_(MOD), or iii) WO_3_/BiVO_4_/Al_2_O_3_(CVD). Time profiles of e) H_2_O_2_ concentration and f) FE for H_2_O_2_ generation in a two‐electrode PEC system using i) WO_3_/BiVO_4_, ii) WO_3_/BiVO_4_/Al_2_O_3_(MOD), or iii) WO_3_/BiVO_4_/Al_2_O_3_(CVD) as the photoanode, a Pt wire cathode, a 2 M aqueous KHCO_3_ solution electrolyte, and a solar simulator light source with a constant current of 1 mA. g) Proposed mechanism for H_2_O_2_ decomposition suppression by adding Al_2_O_3_ layer onto a WO_3_/BiVO_4_ photoanode. MOD and CVD refer to metal–organic decomposition and chemical vapor deposition, respectively, which are the techniques used to deposit Al_2_O_3_ on the WO_3_/BiVO_4_ photoanode. Reproduced with permission.^[^ ^41c^ ^]^ Copyright 2018, Royal Society of Chemistry.

The low FE(H_2_O_2_) observed for the WO_3_/BiVO_4_ photoanode could be attributed to the undesired oxidative degradation of generated H_2_O_2_ to O_2_. To address this issue, the WO_3_/BiVO_4_ photoanode surface was strategically modified with various amorphous, mesoporous metal oxide layers (MeO*_x_*) deposited using the metal–organic decomposition (MOD) method (**Figure** **12**a).^[^ ^41a^ ^]^ Compared to the unmodified WO_3_/BiVO_4_ photoanode, all MeO*_x_*‐coated photoanodes exhibited enhanced H_2_O_2_ generation performance, with the exception of CoO*_x_* (Figure 12b). The observed enhancement followed the trend: CoO*_x_* << SiO_2_ < TiO_2_ < ZrO_2_ < Al_2_O_3._ Notably, the FE(H_2_O_2_) of WO_3_/BiVO_4_ photoanode almost doubled upon the addition of an Al_2_O_3_ surface coating. The subpar performance of CoO*_x_* in H_2_O_2_ generation was attributed to its propensity to catalyze either O_2_ evolution or H_2_O_2_ decomposition reactions. In a 2M KHCO_3_ electrolyte, the FE(H_2_O_2_) was enhanced from *≈*54% for the unmodified WO_3_/BiVO_4_ photoanode to 79% for the Al_2_O_3_‐coated WO_3_/BiVO_4_ photoanode (denoted as WO_3_/BiVO_4_/Al_2_O_3_(MOD)). Additionally, the H_2_O_2_ yield at 50C increased from ≈1300 µM for the unmodified photoanode to ≈2500 µM for the WO_3_/BiVO_4_/Al_2_O_3_(MOD) photoanode. The Al_2_O_3_ layer deposited on the BiVO_4_ surface is speculated to function as a passivation layer, potentially exerting three effects: i) preventing direct contact between BiVO_4_ and the generated H_2_O_2_ that diffuses into the electrolyte, thereby suppressing its oxidative degradation, ii) inhibiting direct O_2_ evolution from four‐electron WOR by covering the active sites, and iii) enriching the local KHCO_3_ concentration around the photoanode due to the acid‐base adsorption between HCO_3_^−^ anions (weak base) and the mildly acidic Al_2_O_3_ surface.

However, one main drawback of the MOD method was the formation of a thick mesoporous Al_2_O_3_ layer on the WO_3_/BiVO_4_ photoanode. This excessively thick Al_2_O_3_ layer could potentially disrupt electrical contact between the BiVO_4_ and the electrolyte solution, as substantiated by the decreased photocurrent density generated by WO_3_/BiVO_4_/Al_2_O_3_(MOD) compared to the unmodified WO_3_/BiVO_4_ photoanode (Figure 12c). Additionally, the ABPE also exhibited a decline, decreasing from 2.2% for the unmodified WO_3_/BiVO_4_ photoanode to 1.5% for the WO_3_/BiVO_4_/Al_2_O_3_(MOD) photoanode (Figure 12d). Chemical vapor deposition (CVD) was subsequently demonstrated as an alternative fabrication method capable to depositing a thin Al_2_O_3_ layer on the WO_3_/BiVO_4_ photoanode.^[^ ^41c^ ^]^ The obtained WO_3_/BiVO_4_/Al_2_O_3_(CVD) not only enhanced the FE(H_2_O_2_) to 80% using a 2M KHCO_3_ electrolyte solution but also maintained its photocurrent generation ability (Figure 12c), leading to an improved ABPE with a maximum value of 2.57% (Figure 12d). Nevertheless, the presence of an Al_2_O_3_ layer (regardless of the fabrication methods) consistently enhanced the H_2_O_2_ yield with respect to the unmodified WO_3_/BiVO_4_ photoanode, as shown in Figure 12e. Analysis of the FE(H_2_O_2_) for all photoanodes over time (Figure 12f) revealed a gradual decrease due to the oxidative degradation of H_2_O_2_ into O_2_. However, the rate of decrease for Al_2_O_3_‐modified photoanodes was found to be slower than that of the unmodified WO_3_/BiVO_4_ photoanode. This indicates that the Al_2_O_3_ layer inhibits the oxidative degradation of generated H_2_O_2_ into O_2_, possibly by blocking H_2_O_2_ interaction with the WO_3_ underlayer (Figure 12g).

**Figure 13:**
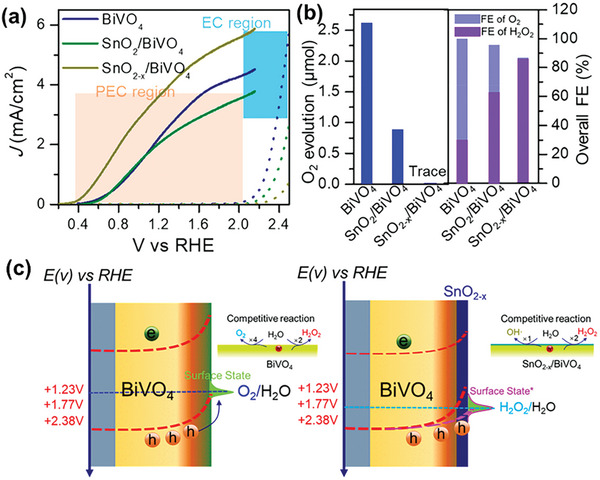
Effect of oxygen‐deficient SnO_2_ (SnO_2‐_ *_x_*) coating on BiVO_4_ photoanode for H_2_O_2_ production. a) *J*–*V* curves and b) O_2_ evolution amount and overall FE values for both H_2_O_2_ and O_2_ evolution at 1.23 V versus RHE from BiVO_4_, SnO_2_/BiVO_4_, and SnO_2‐_ *_x_*/BiVO_4_ photoanodes in 1 M NaHCO_3_ (pH 8.3) electrolyte under AM 1.5G illumination. c) Schematic diagram illustrating the effect of SnO_2‐_ *_x_* on shifting the surface states of the BiVO_4_ photoanode to align with the H_2_O_2_ generation potential, promoting two‐electron and one‐electron WOR for H_2_O_2_ and ^•^OH generation while suppressing four‐electron WOR for O_2_ evolution. Reproduced with permission.^[^ ^43a^ ^]^ Copyright 2020, American Chemical Society.

In contrast, Zhang et al. utilized an oxygen‐deficient SnO_2_ coating (SnO_2‐_ *_x_*) as a passivation layer on the BiVO_4_ photoanode, effectively modulating the surface reaction kinetics to attain highly selective water oxidation for H_2_O_2_ generation.^[^ ^43a^ ^]^ While the SnO_2‐_ *_x_*/BiVO_4_ configuration exhibited enhanced photocurrent density compared to bare BiVO_4_ and SnO_2_/BiVO_4_ photoanodes, it showed an apparent negative shift in the onset potential (**Figure** **13**a). Quantitative analysis of oxidation products indicated that the SnO_2‐_ *_x_*/BiVO_4_ photoanode yielded a negligible amount of O_2_, while BiVO_4_ and SnO_2_/BiVO_4_ photoanodes produced 2.628 and 0.773 µmol of O_2_, respectively (Figure 13b), implying near‐complete suppression of O_2_ evolution (from both water oxidation and H_2_O_2_ decomposition) by the SnO_2‐_ *_x_*/BiVO_4_ photoanode. Additionally, the presence of an SnO_2_ or SnO_2‐_ *_x_* overlayer on the BiVO_4_ photoanode induced the formation of ^•^OH, originating from one‐electron WOR (H_2_O → ^•^OH + H^+^ + e^−^), potentially facilitating H_2_O_2_ generation. Surface photovoltage and flat band potential measurements indicated that the addition of an SnO_2‐_ *_x_* overlayer on BiVO_4_ promoted hole migration to the surface, thus kinetically favoring H_2_O_2_ evolution with high selectivity due to reduced band bending (Figure 13c). Consequently, surface modification of the BiVO_4_ photoanode with an SnO_2‐_ *_x_* overlayer regulates the surface reactions, shifting from the competing hole oxidation reactions of H_2_O_2_ production and O_2_ evolution to favor H_2_O_2_ and ^•^OH generation while also suppressing H_2_O_2_ decomposition. The SnO_2‐_ *_x_*/BiVO_4_ photoanode exhibited an average FE(H_2_O_2_) of 86% over a wide potential range of 0.6–2.1 V versus RHE. At 1.23 V versus RHE, it achieved an average H_2_O_2_ evolution rate of 0.825 µmol min^−1^ cm^−2^ under AM 1.5G illumination, corresponding to an ABPE of *≈*5.6%, surpassing reported values.

**Figure 14:**
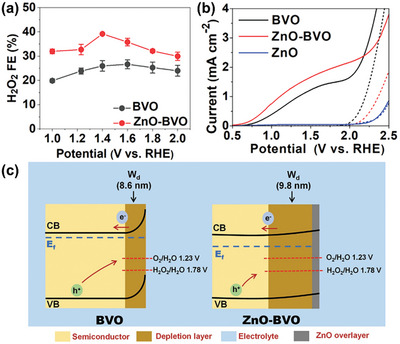
Effect of ZnO coating on BiVO_4_ photoanode for H_2_O_2_ production. a) FE for H_2_O_2_ production on bare BiVO_4_ (BVO) and ZnO‐coated BiVO_4_ (ZnO‐BVO) photoanodes as a function of applied potentials. b) Linear sweep voltammetry (LSV) curves of BVO, ZnO‐BVO, and ZnO photoanodes recorded under AM 1.5G illumination (solid line) or in the dark (dashed line) in 2 M KHCO_3_. c) Schematic illustration of the impact of ZnO on regulating the interfacial energetics of the BiVO_4_ photoanode (BVO) to promote the two‐electron WOR for H_2_O_2_ generation by aligning with its redox potential. Reproduced with permission.^[^ ^44a^ ^]^ Copyright 2023, John Wiley and Sons.

Ng and his team uncovered zinc oxide (ZnO) as another effective passivator for enhancing the selectivity and activity of BiVO_4_ in PEC H_2_O_2_ production.^[^ ^44a^ ^]^ A homogeneous layer of ZnO on the BiVO_4_ photoanode demonstrably improved the FE(H_2_O_2_) within the potential range of 1.0–2.0 V versus RHE under AM 1.5G irradiation, (**Figure** **14**a). The highest FE(H_2_O_2_) of *≈*39.7% was achieved at 1.4 V versus RHE, representing an approximately 1.5‐fold increase compared to the unmodified BiVO_4_ photoanode. Notably, both unmodified and ZnO‐coated BiVO_4_ photoanodes produced O_2_ as the only byproduct. Moreover, the ZnO‐coated BiVO_4_ photoanode exhibited higher photocurrent densities than the unmodified counterpart, accompanied by an evident negative shift in the onset potential (Figure 14b). The thin ZnO passivation layer on the BiVO_4_ photoanode was revealed to flatten the band bending and induce a positive shift in the quasi‐Fermi level within the depletion layer at the semiconductor/electrolyte interface, as evidenced by Mott–Schottky, open‐circuit potentials, and photoelectrochemical impedance spectra measurements. These combined effects promote selective H_2_O_2_ production while impeding the competing O_2_ evolution reaction, as illustrated in Figure 14c. Additionally, the ZnO overlayer suppresses H_2_O_2_ decomposition and serves as holes reservoir under photoexcitation, facilitating expedited charge extraction from BiVO_4_ for the water oxidation reaction.

**Figure 15:**
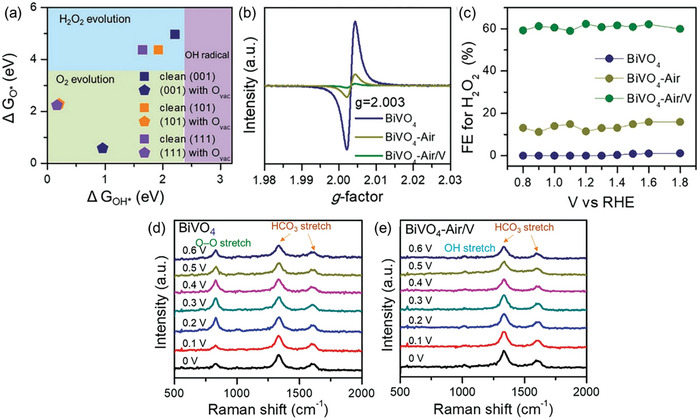
Effect of oxygen vacancies on BiVO_4_ photoanode for H_2_O_2_ production. a) Water oxidation product selectivity map constructed by plotting Δ*G*_O*_ against Δ*G*_OH*_ for three surfaces of BiVO_4_ with and without O_vac_. b) EPR spectra and c) calculated real‐time FE(H_2_O_2_) production on BiVO_4_, BiVO_4_‐Air, and BiVO_4_‐Air/V photoanodes measured in 1M NaHCO_3_ electrolyte (pH 8.3) under AM 1.5G illumination. d,e) In situ Raman spectra of BiVO_4_ and BiVO_4_‐Air/V measured in 1M NaHCO_3_ electrolyte (pH 8.3). Reproduced with permission.^[^ ^51^ ^]^ Copyright 2021, John Wiley and Sons.

Building upon the established effectiveness of surface passivation in enhancing BiVO_4_’s PEC water oxidation for H_2_O_2_ generation, Wang et al. identified intrinsic oxygen vacancies (O_vac_) on the material's surface as a barrier to selective H_2_O_2_ formation.^[^ ^51^ ^]^ While O_vac_ introduction improves electron conductivity and density in n‐type BiVO_4_,^[^ ^58^ ^]^ their roles as traps and recombination sites remain controversial.^[^ ^59^ ^]^ This controversy likely stems from the existence of two types of O_vac_—bulk and surface—with distinct functionalities.^[^ ^60^ ^]^ Wang et al. calculated the Gibbs free energy changes (ΔG) of water oxidation intermediates (OH*, O*, and OOH*) on the most stable (001), (101), and (111) surfaces of BiVO_4_ with and without surface O_vac_. Their calculations (**Figure** **15**a) demonstrated that presence of O_vac_ significantly lowers the activation energy for O_2_ evolution but creates a higher barrier for H_2_O_2_ formation. These results align with their experimental data, where BiVO_4_ photoanodes with reduced surface O_vac_ exhibit superior PEC water oxidative H_2_O_2_ generation. Surface O_vac_ reduction was achieved by post‐treatment with air annealing (BiVO_4_‐Air) and V_2_O_5_‐assisted air annealing (BiVO_4_‐Air/V) as evidenced by weaken EPR signals (Figure 15b). Notably, V_2_O_5_‐rich annealing effectively eliminated the surface O_vac_, as indicated by the negligible EPR signal. Both BiVO_4_‐Air and BiVO_4_‐Air/V photoanodes displayed enhanced photocurrent densities and more cathodic onset potentials compared to the unmodified counterpart. Despite exhibiting a slightly lower photocurrent density (potentially due to lower carrier density), BiVO_4_‐Air/V achieved the highest average FE(H_2_O_2_) of 58.4% within the 0.8–1.8 V versus RHE potential range under AM 1.5G irradiation (Figure 15c). A total amount of 27.7 µmol H_2_O_2_ was produced after 5 h of illumination at 1.23 V versus RHE. In situ Raman analysis using 1M NaHCO_3_ electrolyte identified distinct intermediate species: OOH* for the unmodified photoanode (Figure 15d) and OH* for the BiVO_4_‐Air/V (Figure 15e), further supporting the notion that reduced surface O_vac_ steers the water oxidation pathway toward H_2_O_2_ formation instead of O_2_. Similarly, a more recent study by Qu et al. reported a twofold increase in WOR selectivity for H_2_O_2_ with decreased O_vac_ on a BiVO_4_ photoanode, attributing this improvement to flattened band bending, a positively shifted quasi‐Fermi level, and suppressed H_2_O_2_ decomposition.^[^ ^52^ ^]^ The passivation of O_vac_ was accomplished through thermal treatment of the photoanode in a pressurized Parr reactor filled with O_2_, resulting in the formation of an O‐BiVO_4_ photoanode.

**Figure 16:**
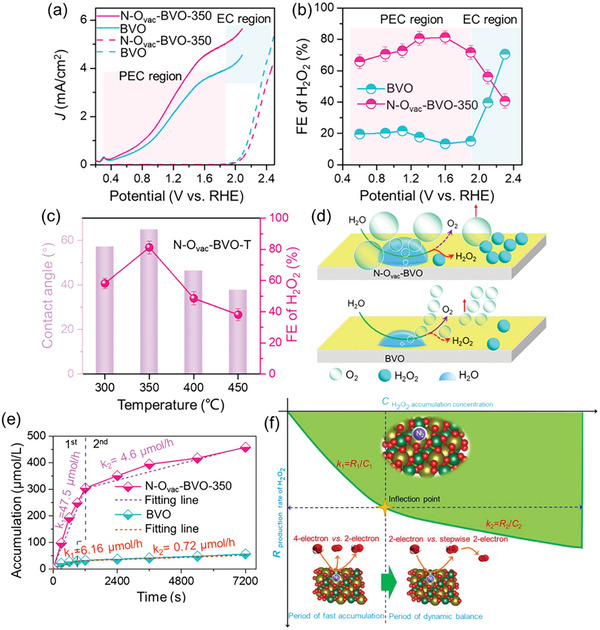
Effect of tuning surface wettability of BiVO_4_ photoanode for H_2_O_2_ production. a) *J*–*V* curves of BVO and N‐O_vac_‐BVO‐350 photoanodes measured under AM1.5G illumination (solid line) or in the dark (dashed line) in a 1M NaHCO_3_ electrolyte. b) Calculated real‐time FE(H_2_O_2_) for various applied biases on BVO and N‐O_vac_‐BVO‐350 photoanodes. c) Correlation between the contact angle and the real‐time FE(H_2_O_2_) for N‐O_vac_‐BVO prepared at different calcination temperatures. d) Schematic diagram illustrating the tuning of surface kinetics of O_2_ evolution and H_2_O_2_ generation rates on BVO and N‐O_vac_‐BVO photoanodes. e) H_2_O_2_ accumulation concentrations on BVO and N‐O_vac_‐BVO‐350 photoanodes measured at 1.6 V versus RHE. f) Schematic diagram depicting the mechanism of H_2_O_2_ accumulation kinetics for the N‐O_vac_‐BVO‐350 photoanode. Reproduced with permission.^[^ ^43b^ ^]^ Copyright 2022, American Chemical Society.

Surface microenvironment modification of BiVO_4_ photoanodes presents a valuable approach to kinetically control of competitive reactions during WOR on the electrode. Specifically, increasing photoanode surface hydrophobicity can inhibit the release of O_2_ while promoting H_2_O_2_ desorption. Wan et al. demonstrated this by post‐treating an oxygen‐vacancy‐enriched BiVO_4_ (O_vac_‐BVO) photoanode, obtained via photoetching, under N_2_ atmosphere at elevated temperature to modulate surface wettability.^[^ ^43b^ ^]^ This N_2_ treatment induced the formation of N_2_ molecules and N atoms binding to O_vac_ sites on BiVO_4_. While the O_vac_ sites were claimed to enhance charge separation, the N species decreased the surface wettability (i.e., increase hydrophobicity) of the as‐obtained N_2_‐treated O_vac_‐BVO photoanode (N‐O_vac_‐BVO) without altering its intrinsic electronic properties (band structure and band bending). The N‐O_vac_‐BVO photoanode with the lowest surface wettability was achieved at a calcination temperature of 350 °C (denoted as N‐O_vac_‐BVO‐350). Compared to the unmodified BiVO_4_ photoanode (BVO), N‐O_vac_‐BVO‐350 exhibited *≈*1.3 and 4.1 times enhancement in photocurrent density (**Figure** **16**a) and average FE(H_2_O_2_) (Figure 16b), respectively, under applied biases of 0.6–1.9 V versus RHE. The H_2_O_2_ production was revealed to be inversely correlated with surface wettability (Figure 16c). As illustrated in Figure 16d, the superior PEC H_2_O_2_ generation performance of N‐O_vac_‐BVO‐350 can be attributed to its poor surface wettability, which effectively increases the surface tension of O_2_ bubbles. This inhibits O_2_ release while promoting H_2_O_2_ separation from the photoanode surface before competitive reactions reach equilibrium, thereby kinetically enhancing H_2_O_2_ production. Nonetheless, H_2_O_2_ accumulated nonlinearly with reaction time for both BVO and N‐O_vac_‐BVO‐350 (Figure 16e), indicating that WOR kinetics for H_2_O_2_ evolution do not follow a first‐order charge transfer mechanism. The initial fast reaction rate likely involves competing four‐electron and two‐electron WOR for O_2_ and H_2_O_2_ generation, respectively, whereas the subsequent slow process relates to competing H_2_O_2_ generation and decomposition reactions (Figure 16f). Illuminating the N‐O_vac_‐BVO‐350 photoanode for 2 h under simulated light and an applied bias of 1.6 V versus RHE yielded a total H_2_O_2_ concentration of 458 µM, corresponding to a production rate of 11.45 µmol h^−1^.

**Figure 17:**
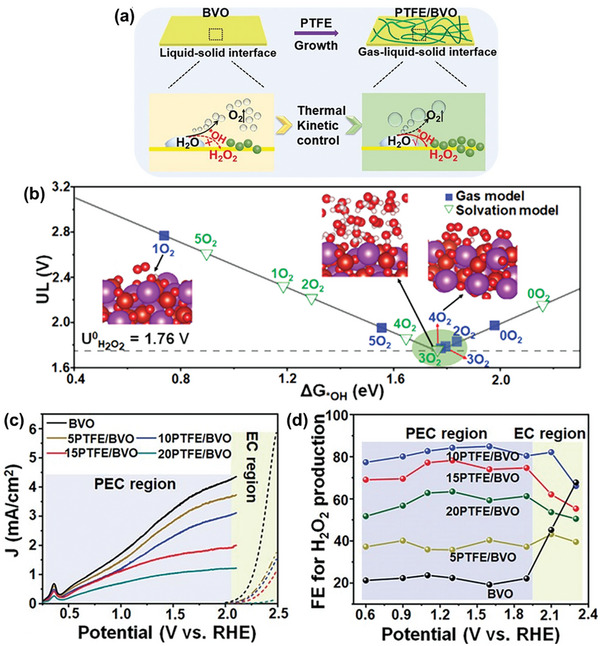
Effect of polytetrafluoroethylene (PTFE) coating on BiVO_4_ photoanode for H_2_O_2_ production. a) Schematic illustration of enhanced H_2_O_2_ production compared to O_2_ evolution by coating a BiVO_4_ photoanode (BVO) with PTFE layer. b) Two‐electron WOR volcano plot as a function of the ΔG_*OH_ over BiVO_4_ covered with O_2_ molecules. c) *J*–*V* curves of BVO and PFTE/BVO photoanodes measured in 1 M NaHCO_3_ electrolyte under AM 1.5G illumination (solid line) or in the dark (dashed line). d) The calculated real‐time FE(H_2_O_2_) of BVO and PFTE/BVO photoanodes at different applied biases. Reproduced with permission.^[^ ^43c^ ^]^ Copyright 2023, John Wiley and Sons.

Similarly, Ou et al. employed surface microenvironment engineering to kinetically favor H_2_O_2_ production over O_2_ evolution. They achieved this by coating the BiVO_4_ photoanode with a thin hydrophobic polytetrafluoroethylene (PTFE) (**Figure** **17**a).^[^ ^43c^ ^]^ This polymer coating is found to confine O_2_ gas near active sites, potentially favoring H_2_O_2_ generation thermodynamically through shifting of *OH intermediates. As depicted in the volcano plot for two‐electron WOR as a function of the *OH binding energy (ΔG_*OH_), particularly within a solvation model, the highest H_2_O_2_ activity can be obtained when Bi sites are surrounded by four O_2_ molecules (Figure 17b). However, the inherent hydrophobicity of PTFE lowers the electrochemical surface area, leading to subpar photocurrent densities for the PTFE‐coated BiVO_4_ photoanodes (PTFE/BVO) compared to the unmodified BiVO_4_ photoanode (BVO) (Figure 17c). Nevertheless, all PTFE/BVO photoanodes exhibited significant enhanced FE(H_2_O_2_) compared to BVO across a wide applied bias range of 0.6 to 2.1 V versus RHE (Figure 17d). Specifically, the highest FE(H_2_O_2_) of 85% was exhibited by the 10PTFE/BVO photoanode, corresponding to a fourfold increase over the BVO photoanode. A final H_2_O_2_ concentration of 150 µM was obtained at 1.23 V versus RHE under AM 1.5G illumination for 2 h.

**Figure 18:**
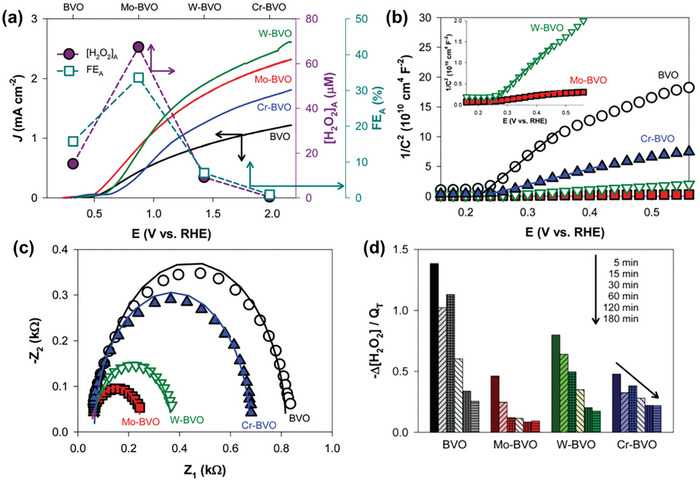
Effect of molybdenum (Mo), tungsten (W), and chromium (Cr) doping on BiVO_4_ photoanode for H_2_O_2_ production. a) LSV curves for BVO, Mo‐BVO, W‐BVO, and Cr‐BVO photoanodes measured under AM 1.5G illumination in a 1 M NaHCO_3_ electrolyte. The concentration of produced H_2_O_2_ ([H_2_O_2_]_A_) and the corresponding FE(H_2_O_2_) were measured after a 15‐min reaction at 1.0 V versus RHE. b) Mott–Schottky plots and c) Nyquist plots of the photoanodes. d) The amount of H_2_O_2_ decomposed by a hole‐mediated reaction divided by the total charge passing through the circuit (*Q*_T_) for all photoanodes as a function of time, with an initial H_2_O_2_ amount of 1 mM. The measurements were conducted at 1.0 V versus RHE under AM 1.5G illumination. Reproduced with permission.^[^ ^53^ ^]^ Copyright 2020, Royal Society of Chemistry.

Doping presents another viable strategy for enhancing the PEC performance of BiVO_4_. Typically, this process involves substituting V^5+^ sites with hexavalent metal ions such as Mo^6+^ and W^6+^ to increase donor density, thereby enhancing electrical conductivity.^[^ ^61^ ^]^ Jeon et al. investigated the impact of various dopants (Mo, W, and Cr) on the PEC H_2_O_2_ production performance of BiVO_4_ photoanodes.^[^ ^53^ ^]^ As shown in **Figure** **18**a, the introduction of these metal dopants enhanced BiVO_4_’s photocurrent generation. This improvement is attributed to the higher electrical conductivity and lower charge transfer resistance observed in BiVO_4_ upon doping, as evidenced by Mott–Schottky measurements (Figure 18b) and Nyquist plots (Figure 18c). Nevertheless, while Mo‐doping improved H_2_O_2_ production and FE(H_2_O_2_), these parameters were compromised with W and Cr doping (Figure 18a). The superior performance of Mo as a dopant for enhancing H_2_O_2_ generation efficiency in BiVO_4_ photoanodes stems from its ability to inhibit H_2_O_2_ decomposition. This is supported by the observation that Mo‐doped BiVO_4_ (Mo‐BVO) exhibited the least amount of H_2_O_2_ decomposition relative to the total charge passing through the PEC cell (–Δ[H_2_O_2_]/Q_T_) during the irradiation duration (3 h) compared to unmodified BiVO_4_ (BVO), W‐doped BiVO_4_ (W‐BVO), and Cr‐doped BiVO_4_ (Cr‐BVO) photoanodes (Figure 18d).

**Figure 19:**
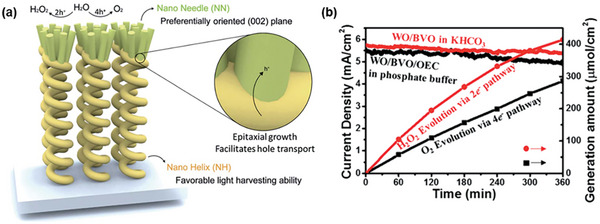
PEC H_2_O_2_ generation performance of carrot‐shaped WO_3_/Mo‐Doped BiVO_4_ photoanode. a) Schematic illustration of the carrot‐shaped WO_3_ NN/NH nanostructure, where the NNs epitaxially grow on top of the NHs. b) Time profiles of photocurrent densities and the amounts of H_2_O_2_ and O_2_ generated by WO/BVO and FeOOH/NiOOH‐loaded WO/BVO (WO/BVO/OEC) photoanodes measured at 0.8 V versus RHE under solar simulator irradiation. 2 M KHCO_3_ and phosphate buffer solutions were used as the respective electrolytes for WO/BVO and WO/BVO/OEC. Reproduced with permission.^[^ ^54^ ^]^ Copyright 2018, Royal Society of Chemistry.

Utilizing a WO_3_/Mo‐doped BiVO_4_ heterojunction photoanode, Shi et al. introduced a unique method to enhance its light absorption and charge carrier dynamics by modulating the WO_3_ core surface before Mo‐doped BiVO_4_ shell formation. In particular, exposing the preformed WO_3_ nanohelices (NHs) on an FTO substrate to a flame surface treatment in a reducing environment created an epitaxial layer of 1D oriented WO_3_ nanoneedles (NNs), as illustrated in **Figure** **19**a.^[^ ^54^ ^]^ The resulting carrot‐shaped WO_3_ NN/NH nanostructure exhibited improved PEC performance compared to the WO_3_ NH counterpart, attributed to changes in optical and electronic properties (i.e., band gap energy, flat band potential, and VB position). Subsequently, the WO_3_ NN/NH nanostructure was employed as a scaffold to form a heterojunction by coating the surface with Mo‐doped BiVO_4_, forming the core–shell WO/BVO photoanode. The obtained WO/BVO photoanode demonstrated activity for both H_2_O_2_ generation and O_2_ evolution in the respective KHCO_3_ and phosphate buffer electrolytes (Figure 19b). For H_2_O_2_ production, ≈414 µmol cm^−2^ of H_2_O_2_ was detected after illuminating the WO/BVO photoanode in 2 M KHCO_3_ for 6 h using a solar simulator at 0.8 V versus RHE. This corresponds to an H_2_O_2_ generation rate of 1.15 µmol min^−1^ cm^−2^.

**Figure 20:**
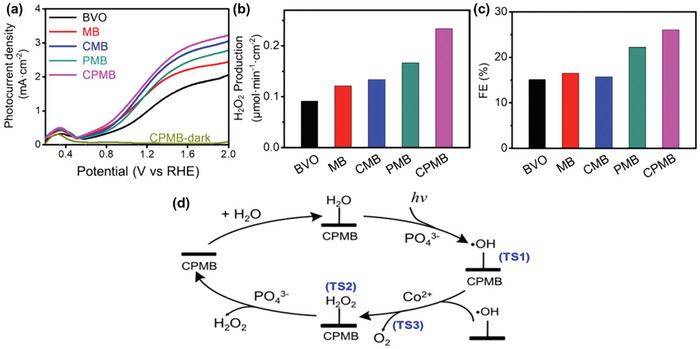
Effect of Co^2+^ and PO_4_^3−^ ions deposition on Mo‐doped BiVO_4_ photoanode for H_2_O_2_ production. a) *J*–*V* curves of BVO, MB, CMB, PMB, and CPMB measured in 2M KHCO_3_ solution under AM 1.5G illumination. b) The real‐time H_2_O_2_ production rate and c) FE(H_2_O_2_) obtained using the photoanodes at 1.7 V versus RHE. d) Schematic illustration of the proposed reaction mechanism for H_2_O_2_ production on CPMB photoanode. Reproduced with permission.^[^ ^41d^ ^]^ Copyright 2022, Elsevier.

Yang et al. demonstrated that co‐deposition of Co^2+^ and PO_4_^3−^ ions (CPMB) onto the Mo‐doped BiVO_4_ (MB) surface constructively improves their activity for PEC water oxidative H_2_O_2_ generation via ^•^OH radical intermediates.^[^ ^41d^ ^]^ While modifying MB with either Co^2+^ or PO_4_^3−^ ions alone (denoted as CMB and PMB, respectively) improved photocurrent density generation compared to unmodified BiVO_4_ (BVO) and MB photoanodes, a synergistic effects was realized with the co‐deposition of both ions, resulting in further enhancement (**Figure** **20**a). At 1.7 V versus RHE, the H_2_O_2_ production activity trend followed the order: BVO < MB < CMB < PMB < CPMB (Figure 20b). The CPMB photoanode achieved an optimal H_2_O_2_ evolution rate of 0.23 µmol min^−1^ cm^−2^ with a FE(H_2_O_2_) of 26% (Figure 20c). In situ EPR spectroscopy, revealed the distinct roles of Co^2+^ and PO_4_^3−^ ions in contributing to the improved H_2_O_2_ evolution activity of CPMB (Figure 20d). While PO_4_^3−^ ions promote ^•^OH formation from H_2_O dissociation and H_2_O_2_ desorption from the photocatalyst surface, Co^2+^ ions facilitate the ^•^OH conversion for H_2_O_2_ and O_2_ production. Importantly, Co^2+^ ion plays a vital role in improving the kinetics of both H_2_O_2_ production and O_2_ evolution, supported by CMB showing a comparable FE(H_2_O_2_) to MB and BVO despite a higher H_2_O_2_ yield.

**Figure 21:**
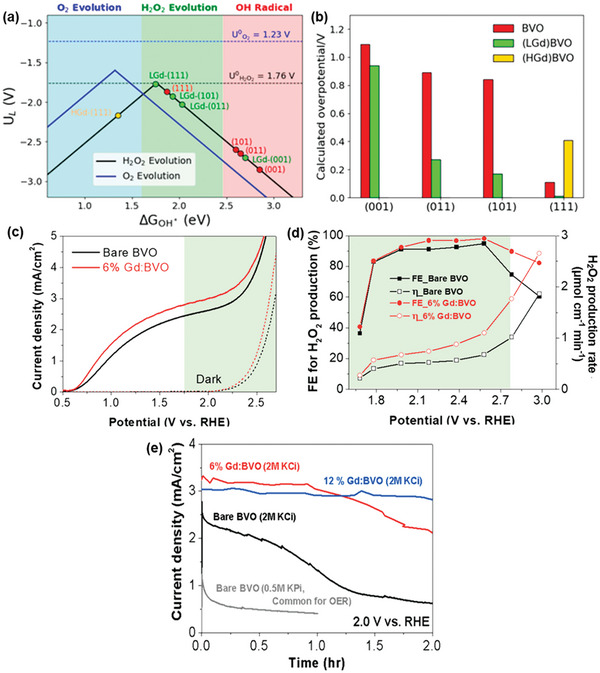
Effect of gadolinium (Gd) doping on BiVO_4_ photoanode for H_2_O_2_ production. a) Volcano plots for two‐electron and four‐electron pathways of water oxidation to produce H_2_O_2_ and O_2_ as a function of the OH* adsorption energies on different facets of BiVO_4_ with or without Gd‐doping. LGd and HGd represent low and high Gd‐doping concentrations. b) The theoretically calculated overpotential required for H_2_O_2_ generation from different facets of BiVO_4_. c) *J*–*V* curves and d) real‐time H_2_O_2_ yield and FE(H_2_O_2_) for bare BiVO_4_ (BVO) and 6% Gd‐doped BiVO_4_ (Gd:BVO) photoanodes measured under 1 sun illumination in 2 M KHCO_3_ (pH ≈ 8.3). The FE(H_2_O_2_) was measured after a 5min reaction at each potential. e) Stability test for 12% Gd:BVO, 6% Gd:BVO, and bare BVO in 2 M KCl electrolyte, along with bare BVO in 0.5 M KPi electrolyte, conducted for 2 h at 2.0 V versus RHE under 1 sun illumination. Reproduced with permission.^[^ ^55^ ^]^ Copyright 2019, American Chemical Society.

Molybdenum remains the most prevalent and effective dopant for BiVO_4_. However, a recent study from Baek et al. explored gadolinium (Gd), a rare earth element, as a dopant to reduce the overpotential for water oxidative H_2_O_2_ production in BiVO_4_ photoanodes, while also improving its stability.^[^ ^55^ ^]^ Gd was chosen due to its elemental stability, good electrical conductivity, and strong oxygen affinity compared to bismuth (Bi). This stronger oxygen affinity is anticipated to inhibit VO_4_^3−^ anion dissolution in BiVO_4_, a critical stability issue under both dark and illuminated conditions.^[^ ^62^ ^]^ According to the Bi‐V Pourbaix diagram calculated by Toma et al., BiVO_4_ is stable across a wide pH range of 1–11, but V dissolution into VO_4_^−^ ions is predicted to occur near the water oxidation potential. This dissolution is accelerated by illumination and anodic biasing to 1.23 V versus RHE.^[^ ^62b^ ^]^ Baek et al.’s DFT calculations revealed that doping a low concentration of Gd can activate several inactive BiVO_4_ facets (i.e., (011) and (101)) for H_2_O_2_ production, as illustrated in **Figure** **21**a. The calculations also predicted a decrease in the overpotential required for H_2_O_2_ production on different facets of Gd‐doped BiVO_4_ compared to bare BiVO_4_ (Figure 21b). Additionally, the estimated increase in the energy barrier for VO_4_^3−^ dissolution by 0.65 eV suggests improved stability for Gd‐doped BiVO_4_. Experimental results corroborated these theoretical findings. With an optimal Gd doping concentration of 6%, the overpotential for H_2_O_2_ production reduced by *≈*110 mV compared to pristine BiVO_4_ under dark conditions. Under 1 sun illumination, the Gd‐doped BiVO_4_ photoanode portrayed a negatively shifted photocurrent onset, higher photocurrent densities, higher FE(H_2_O_2_), and an improved H_2_O_2_ production rate (Figure 21c,d). The Gd‐doped BiVO_4_ photoanode accomplished an FE(H_2_O_2_) of *≈*99.5% at 2.6 V versus RHE. The improved stability resulting from Gd doping is evident from the sustained photocurrent densities exhibited for the Gd‐doped BiVO_4_ photoanodes compared to the pristine BiVO_4_ in the stability test (Figure 21e).

**Figure 22:**
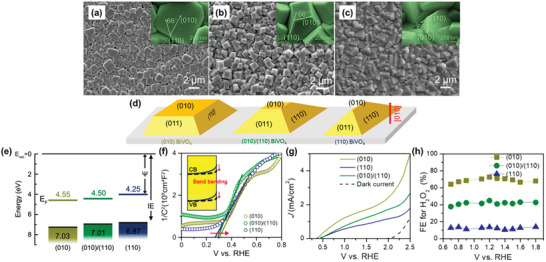
Effect of BiVO_4_ crystal facet on PEC performance for H_2_O_2_ production. a–c) SEM images of the (010), (010)/(110), and (110) facet‐terminated BiVO_4_ photoanodes. d) The corresponding schematic morphologies of the BiVO_4_ microcrystals on the BiVO_4_ photoanodes. e) Band structures of the three photoanodes constructed based on UPS results. f) Mott–Schottky plots, g) *J*–*V* curves, and h) real‐time FE(H_2_O_2_) of the three photoanodes measured in 1 M NaHCO_3_ under AM 1.5G illumination. Reproduced with permission.^[^ ^43e^ ^]^ Copyright 2021, American Chemical Society.

Crystal facet engineering, particularly the fabrication of BiVO_4_ with exposed (010) and (110) facets, has emerged as a promising strategy to improve charge separation due to the differing band energy levels between the facets. Park et al. provided new insights into how crystal facet engineering can be used to manipulate interfacial energetics (i.e., band bending and quasi‐Fermi level) at the BiVO_4_/electrolyte interface, thereby regulating the product selectivity for H_2_O_2_ or O_2_ during water oxidation in a PEC system.^[^ ^43e^ ^]^ Three BiVO_4_ photoanodes with varying ratios of exposed (010) and (110) facets were synthesized using a controlled hydrothermal method (**Figure** **22**a–c). The morphologies of the BiVO_4_ microcrystals on each photoanode are schematically depicted in Figure 22d. The photoanodes are designated as (010), (010)/(110), and (110) based on their dominant exposed facets. Ultraviolet photoelectron spectroscopy (UPS) analysis revealed significant differences in the band energy levels between the three BiVO_4_ photoanodes (Figure 22e). Specifically, the work function and VB maxima shifted away from the vacuum level with increasing (010) facet exposure. This indicates a decreasing Fermi level and less upward band bending. The weaker band bending leads to a more anodic hole quasi‐Fermi level, which was also attested with the positive shift of flat‐band potential observed in Mott–Schottky plots (Figure 22f). A more anodic hole quasi‐Fermi level was hypothesized to increase the oxidation potential of holes to favor water oxidation at a higher potential level, for instances water oxidative H_2_O_2_ production at 1.77 V versus RHE is preferred over water oxidative O_2_ evolution at 1.23 V versus RHE. Such hypothesis is testified with improved photocurrent densities and a negative shift in onset potentials for BiVO_4_ photoanodes with increasing (010) facet (Figure 22g). The average FE(H_2_O_2_) was calculated to enhance from 11% for the (110) photoanode to 70% for the (010) photoanode at applied bias ranging from 0.6 to 1.8 V versus RHE under AM 1.5G illumination (Figure 22h). Using the (010) photoanode, the ABPE for PEC water oxidation to H_2_O_2_ was found to be 30 times higher compared to O_2_ evolution.

While the focus thus far has been on the design and development of BiVO_4_ photoanodes for enhanced PEC water oxidative H_2_O_2_ generation, electrolyte selection presents another approach to influence catalytic activity and selectivity as it plays a crucial role in charge migration and mass diffusion within the system. Concentrated HCO_3_^−^ electrolyte is commonly acknowledged as the most effective reaction medium for directly producing H_2_O_2_ from water oxidation. However, recent studies indicate that HCO_3_^–^ can deplete the generated H_2_O_2_ through unwanted reactions.^[^ ^63^ ^]^

**Figure 23:**
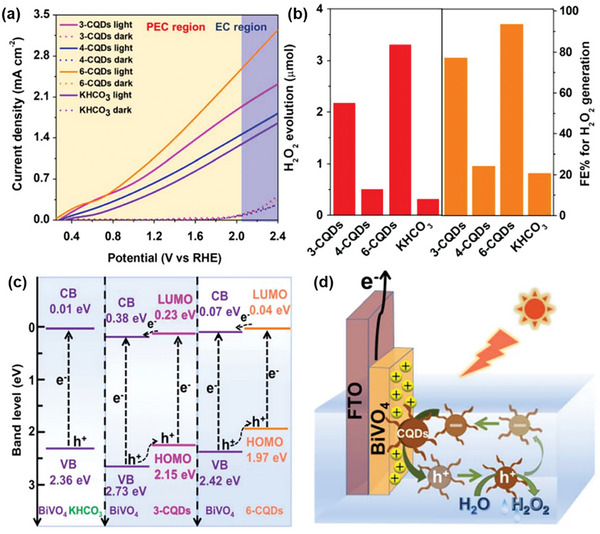
PEC H_2_O_2_ generation from carbon quantum dots (CQDs) aqueous solution. a) *J*–*V* curves of the BiVO_4_ photoanode in CQDs aqueous solutions and KHCO_3_ electrolyte under 420 nm LED lamp illumination (solid line) or in the dark (dashed line). b) The corresponding yield of H_2_O_2_ and FE(H_2_O_2_) at 1.23 V versus RHE. c) The estimated BiVO_4_ band potentials in KHCO_3_ electrolyte and CQDs aqueous solutions. d) Schematic illustration of the proposed mechanism of water oxidative H_2_O_2_ production on the BiVO_4_ photoanode in CQDs aqueous solution. Reproduced with permission.^[^ ^43d^ ^]^ Copyright 2023, Royal Society of Chemistry.

Wang et al. highlighted the potential of carbon quantum dots (CQDs) aqueous solution as an alternative electrolyte.^[^ ^43d^ ^]^ They prepared three CQDs aqueous solutions (3‐CQDs, 4‐CQDs, and 6‐CQDs) using different linear aliphatic amino acids as precursors. Interestingly, illuminating the BiVO_4_ photoanode with a 420 nm LED lamp in the CQDs solutions enhanced photocurrent densities across a wide range of applied biases compared to the traditional KHCO_3_ electrolyte (**Figure** **23**a). Furthermore, the CQDs solutions yielded H_2_O_2_ production amounts surpassing KHCO_3_ (Figure 23b). The 6‐CQDs solution demonstrated the best performance, producing H_2_O_2_ at an average rate of 0.33 µmol min^−1^ cm^−2^ with an FE(H_2_O_2_) of 93.5% at 1.23 V versus RHE. The superior performances of the CQDs solutions can be attributed to several factors: i) improved charge separation due to the formation of a dynamic heterojunction between the BiVO_4_ substrate and CQDs particles (Figure 23c); ii) the hydrophilic surface of CQDs allows water molecule confinement, facilitating water oxidation to H_2_O_2_ via ^•^OH radicals formation; and iii) efficient inhibition of H_2_O_2_ decomposition. Figure 23d illustrates the proposed mechanism of PEC water oxidation to H_2_O_2_ using a BiVO_4_ photoanode with a CQDs solution. Due to an accumulation of photogenerated holes at the positively charged BiVO_4_ surface, negatively charged CQDs particles can be attracted via electrostatic attraction. Subsequently, they are oxidized by the holes and released back into the solution to drive water oxidation for H_2_O_2_ production before restoring to their original states. This adsorption and desorption process of CQDs on the BiVO_4_ surface continues until an equilibrium is achieved.

#### 4.2.2 Bias‐Free, Dual‐Sided H_2_O_2_ Generation

**Figure 24:**
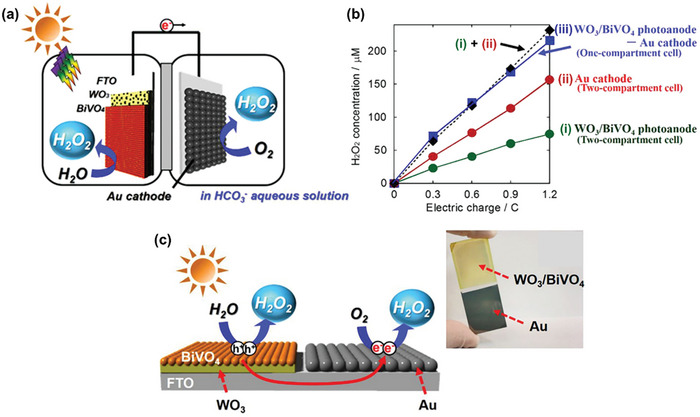
Bias‐free PEC production of H_2_O_2_. a) Schematic diagram of the bias‐free PEC system constituting WO_3_/BiVO_4_ as the photoanode, Au as the cathode, and aqueous KHCO_3_ solution as the electrolyte for H_2_O_2_ production on both electrodes. b) Concentrations of H_2_O_2_ generated via i) H_2_O oxidation on a WO_3_/BiVO_4_ photoanode, ii) O_2_ reduction on an Au cathode within a two‐compartment cell, and iii) simultaneous H_2_O oxidation and O_2_ reduction on the respective WO_3_/BiVO_4_ photoanode and Au cathode within a one‐compartment cell in a 2 M KHCO_3_ aqueous solution under simulated light irradiation. c) Image of a composite electrode consisting of WO_3_/BiVO_4_ and Au on a single FTO substrate, with the reaction mechanism shown on the left. Reproduced with permission.^[^ ^41b^ ^]^ Copyright 2017, John Wiley and Sons.

When utilizing BiVO_4_ as the photoanode, an external bias is necessary to induce H_2_ generation on the counter electrode. This is because the CB potential of BiVO_4_ is more positive than the standard reduction potential for converting H_2_O to H_2_ (Equation 7). Conversely, the standard reduction potential for reducing O_2_ to H_2_O_2_ (Equation 1) is more positive than the CB potential of BiVO_4_, indicating the feasibility of H_2_O_2_ production at the counter electrode even in the absence of an external bias. Sayama's team demonstrated this concept by assembling a PEC system with an Au cathode, a WO_3_/BiVO_4_ photoanode, and a 2 M aqueous KHCO_3_ solution electrolyte.^[^ ^41b^ ^]^ Under simulated solar light and without an external bias, this system showcased H_2_O_2_ generation on both electrodes (**Figure** **24**a). While H_2_O is oxidized to H_2_O_2_ at the anode, the cathode simultaneously produces H_2_O_2_ through O_2_ reduction, realizing a dual‐sided H_2_O_2_ generation process. In a two‐compartment cell, calculations revealed FE(H_2_O_2_) of *≈*50% and *≈*90% for the photoanode and cathode, respectively. Conspicuously, comparable H_2_O_2_ production was yielded in a one‐compartment cell (Figure 24b), indicating that the ion‐exchange membrane is dispensable. The researchers further demonstrated light‐triggered simultaneous H_2_O_2_ generation using single photocatalyst sheet, which consisted of one half coated with WO_3_/BiVO_4_ and the other half coated with Au on an FTO substrate (Figure 24c). Exposing this sheet to simulated sunlight for 60 min in 2 M KHCO_3_ electrolyte reportedly generated 130 µM of H_2_O_2_. The team went on to showcase another bias‐free PEC system consisting of a WO_3_/BiVO_4_/Al_2_O_3_(CVD) photoanode and a biomass‐derived carbon (WSoy/GnP‐CP) cathode. This combination simultaneously generated H_2_O_2_ with FE(H_2_O_2_) values of 60% and 44%, respectively.^[^ ^41c^ ^]^ The combination also showed an open‐circuit voltage of *≈*0.30 V and a short‐circuit current density of 0.7 mA.

Shi et al. underlined such a bias‐free, unassisted PEC system to be a light‐driven fuel cell that utilizes light to trigger H_2_O_2_ production at both electrodes in the presence of water and oxygen, while concurrently generating electricity.^[^ ^56^ ^]^ The simplified reaction can be expressed as follows:

(19) $$\mathrm{light}+2H_{2}O+O_{2}=\mathrm{electricity}+2H_{2}O_{2}$$

Assuming that a BiVO_4_ photoanode absorbs all the photons with energies greater than its bandgap (2.4 eV) and all the photogenerated charge carriers are fully utilized for H_2_O_2_ generation (i.e., 100% internal quantum efficiency and 100% Faraday efficiency), a theoretical maximum H_2_O_2_ production rate of 4.6 µmol min^−1^ cm^−2^ can be attained under 1 sun irradiation. The team proceeded to investigate the effects of electrolyte composition and O_2_ purging on H_2_O_2_ production activity of both the BiVO_4_ photoanode and the carbon‐supported carbon paper cathode (C‐cathode) in their bias‐free PEC system. Consistent with previously studies,^[^ ^22^, ^24^ ^]^ the BiVO_4_ photoanode presented superior H_2_O_2_ production performance in a 2 M KHCO_3_ electrolyte compared to a 1 M Na_2_SO_4_ electrolyte, achieving a maximum FE(H_2_O_2_) of 95% at 1.7 V versus RHE. This can be ascribed to the role of HCO_3_^−^ anion as a catalyst in H_2_O_2_ production. The valence band (VB) potential of BiVO_4_ (*≈* +2.4 V versus NHE)^[^ ^41b^ ^]^ is thermodynamically sufficient for driving HCO_3_^−^ oxidation to HCO_4_^−^ (Equation 20). Subsequently, HCO_4_^−^ acts as a mediator, oxidizing H_2_O to H_2_O_2_ while regenerating HCO_3_^−^ (Equation 21).

(20) $$\begin{array}{ccc} \mathrm{HC}{O_{3}}^{-} & & +H_{2}O↔\mathrm{HC}{O_{4}}^{-}+2H^{+}+2e^{-}\;\;E^{\circ }\left(\mathrm{HC}{O_{4}}^{-}/\mathrm{HC}{O_{3}}^{-}\right)\\ & & =+0.18V\;\mathrm{versus}\;\mathrm{NH}E^{\left[41b\right]} \end{array}$$

(21) $$\mathrm{HC}{O_{4}}^{-}+H_{2}O↔H_{2}O_{2}+\mathrm{HC}{O_{3}}^{-}\;\;$$

On the contrary, the cathode exhibited the opposite trend, with a higher FE(H_2_O_2_) observed in the 1 M Na_2_SO_4_ electrolyte compared to the 2 M KHCO_3_ electrolyte. This discrepancy might be attributed to the abundance of electrons at the cathode surface, which can drive Equation (20) in the reverse direction. The resulting higher concentration of HCO_3_^−^ leads to the consumption of H_2_O_2_ through the reverse process of Equation (21), ultimately leading to lower H_2_O_2_ production when KHCO_3_ is used.

**Figure 25:**
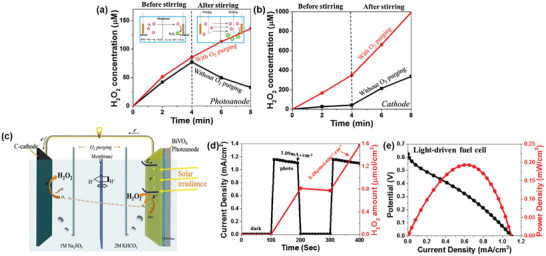
Optimization of H₂O₂ production in a light‐driven fuel cell. Effects of O_2_ purging on H_2_O_2_ production at the a) BiVO_4_ photoanode and b) C‐cathode, conducted under 1 sun irradiation. c) Schematic diagram of the design of a light‐driven fuel cell for H_2_O_2_ generation under optimal operating conditions. d) Short‐circuit current density and amount of H_2_O_2_ generated by the light‐driven fuel cell without an external bias and under light‐on and ‐off conditions. e) *J*–*V* and calculated power density curves of the light‐driven fuel cell. Reproduced with permission.^[^ ^56^ ^]^ Copyright 2018, John Wiley and Sons.

While O_2_ serves as a crucial reactant for H_2_O_2_ production via the ORR at the cathode, it is an undesirable product of the oxygen evolution reaction at the photoanode. Interestingly, O_2_ purging was found to be essential to promote H_2_O_2_ production at both the photoanode and cathode. As shown in **Figure** **25**a, O_2_ purging has an insignificant impact on the H_2_O_2_ yield in the absence of mechanical stirring, as O_2_ diffusion between the electrodes is slow. However, when stirring is introduced, H_2_O_2_ production with O_2_ purging continues to rise, whereas it declines without it. This decline occurs because, under stirring and without O_2_ purging, O_2_ produced at the photoanode is transported to the cathode and consumed by the ORR. This, in turn, promotes the consumption of H_2_O_2_ at the photoanode to replenish O_2_ for the OER. In contrast to the photoanode, O_2_ purging consistently increases the H_2_O_2_ yield at the cathode regardless of stirring (Figure 25b). This is because O_2_ acts as the essential reactant for H_2_O_2_ production.

Building upon the optimal operating conditions identified, the researchers constructed a PEC system for obtaining dual‐sided H_2_O_2_ production on both electrodes. This system comprised a BiVO_4_ photoanode with 2 M KHCO_3_ electrolyte, a C‐cathode with 1 M Na_2_SO_4_ electrolyte, separated by a membrane, and employed O_2_ purging on both sides (Figure 25c). H_2_O_2_ production was achieved using this system under both biased and unbiased conditions. Under an external bias of 1.5 V applied between the two electrodes, the optimized PEC setup produced *≈*2610 ppm and 1530 ppm of H_2_O_2_ at the cathode and photoanode, respectively, after a 5 h reaction. This corresponds to a net production rate of 2.42 µmol min^−1^ cm^−2^. Interestingly, the study also demonstrated the potential of this setup to generate water disinfection‐grade H_2_O_2_ using both tap water and distilled water. The system produced a total of 497 ppm and 319 ppm H_2_O_2_ using tap water and distilled water, respectively. In the absence of an external bias, the optimized system was still able to produce H_2_O_2_ at a rate of 0.48 µmol min^−1^ cm^−2^ (0.18 µmol min^−1^ cm^−2^ for photoanode and 0.30 µmol min^−1^ cm^−2^ for cathode) (Figure 25d). The system had an open‐circuit voltage of 0.61 V and a short‐circuit current density of 1.09 mA cm−^2^ (Figure 25e). The maximum output power density was reported as 0.194 mW cm^−2^.

**Figure 26:**
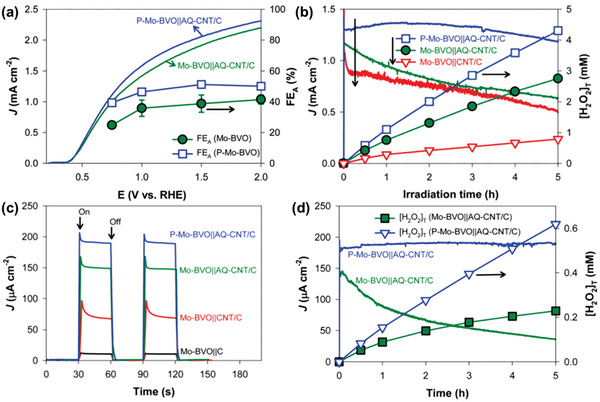
Dual‐sided PEC H₂O₂ generation using Mo‐BVO or P‐Mo‐BVO photoanode. a) LSV curves for the performance of the P‐Mo‐BVO||AQ‐CNT/C and Mo‐BVO||AQ‐CNT/C setups for dual‐sided PEC H_2_O_2_ generation. The anodic FE(H_2_O_2_) (FE_A_) was measured after a 15‐min reaction at each potential. b) Time profiles of the photocurrent density and generated H_2_O_2_ concentration of the P‐Mo‐BVO||AQ‐CNT/C, Mo‐BVO||AQ‐CNT/C, and Mo‐BVO||CNT/C configurations, recorded in 1M NaHCO_3_ solution (pH ≈ 7.8) under AM 1.5G irradiation at 1.0 V versus RHE. c) Short‐circuit current densities of the various photoanode||cathode configurations under light‐on and ‐off conditions. d) Time profiles of bias‐free photocurrent density and H_2_O_2_ generation for the P‐Mo‐BVO||AQ‐CNT/C and Mo‐BVO||AQ‐CNT/C configurations in 1M NaHCO_3_ solution (pH ≈ 7.8) under AM 1.5G irradiation. Reproduced with permission.^[^ ^53^ ^]^ Copyright 2020, Royal Society of Chemistry.

Jeon et al. discovered that anthraquinone‐modified single‐walled carbon nanotube (AQ‐CNT) is a promising material for achieving highly selective cathodic ORR to produce H_2_O_2_.^[^ ^53^ ^]^ They fabricated an AQ‐CNT/C cathode using carbon paper as the substrate for the AQ‐CNTs. When paired with a Mo‐doped BiVO_4_ photoanode (Mo‐BVO as previously described in Figure 18), this cathode achieved near 100% FE(H_2_O_2_) across a broad bias range (0.75 to 2 V versus RHE) with a negligible amount of H_2_ being detected. However, the Mo‐BVO photoanode itself only achieved a concurrent FE(H_2_O_2_) of 20–40% (**Figure** **26**a), with noticeable amounts of O_2_ produced. Post‐surface treatment of the Mo‐BVO photoanode with phosphate (P‐Mo‐BVO) effectively boosted its FE(H_2_O_2_) to 40–50%. This treatment also improved photocurrent generation and slowed down H_2_O_2_ decomposition. In addition, the P‐Mo‐BVO photoanode was highly durable, maintaining 90% of its photocurrent over a 100‐h reaction period. The higher stability of P‐Mo‐BVO with respect to Mo‐BVO is further evident in time‐profiled photocurrent generation experiments (Figure 26b), conducted at 1 V versus RHE with an AQ‐CNT/C cathode. Clearly, the P‐Mo‐BVO||AQ‐CNT/C configuration depicted a steadier photocurrent than that of the Mo‐BVO||AQ‐CNT/C over a 5 h reaction. Under bias‐free conditions, the P‐Mo‐BVO||AQ‐CNT/C system could generate the highest short‐circuit current density of *≈*0.19 mA under AM 1.5G light irradiation (Figure 26c). Furthermore, this system displayed steady photocurrent generation and H_2_O_2_ production on both electrodes under bias‐free conditions for 5 h (Figure 26d). The net H_2_O_2_ production rate was 0.16 µmol min^−1^ cm^−2^, corresponding to a solar‐to‐H_2_O_2_ conversion efficiency of 0.27%.

## 5 Conclusion and Outlook

Artificial photosynthesis presents a promising avenue for H_2_O_2_ production owing to its safety, economic viability, environmentally benignity, and long‐term sustainability. It utilizes water, oxygen, and solar energy as primary resources, offering a clean and renewable approach. In this review, we evaluate the recent advancements of BiVO_4_‐based materials in this field, with a focus on their applications in both PS and PEC systems, which are pivotal methods in artificial photosynthesis. A comprehensive analysis is provided, encompassing fundamental principles, performance assessment methodologies, photocatalyst and photoelectrode development, and optimization of reaction conditions specific to each system.

Despite BiVO_4_’s promising properties, its performance is hindered by inherent limitations such as poor charge carrier separation and mobility. To mitigate these challenges, various material design strategies have been explored, including doping, heterojunction formation, cocatalyst addition, crystal facet engineering, and surface passivation. These strategies are applied in both PS and PEC systems to enhance BiVO_4_’s photocatalytic activity for H_2_O_2_ generation. However, their implementation differs between the two systems, as detailed in **Table** **4**. While doping and heterojunction formation are common strategies for both, other approaches are specifically tailored to meet the unique requirements of each system, highlighting the nuanced strategies necessary to optimize BiVO_4_’s efficiency for H_2_O_2_ production.

**Table 4:** Comparison of strategies for material modifications and reaction conditions in H_2_O_2_ production using PS and PEC systems.

Category	Strategy/Condition	Powder Suspension (PS)	Photoelectrochemical (PEC)
Material Modification Strategies	Doping	Enhances bulk conductivity and material stability	Enhances bulk conductivity and material stability
	Heterojunction	Improves charge separation by incorporating another photocatalyst	Improves charge separation by incorporating another photocatalyst
	ORR and WOR cocatalysts	Provides active sites and enhances surface reaction kinetics	Not applicable
	Crystal facet Engineering	Enables spatial charge separation on different facets	Not applicable
	Surface passivation	Not applicable	Optimizes BiVO_4_/electrolyte interfacial reaction kinetics and selectivity for H_2_O_2_ production
Reaction Conditions	O_2_ atmosphere	Required	Not required
	CO_2_ bubbling	Not required	Required (beneficial for anode reaction)
	Temperature control	Preferred (room temperature or lower)	Not necessary
	Sacrificial reagent	Preferred	Not necessary
	Reaction medium	Pure water	Bicarbonate solution (HCO_3_^−^)

The design of BiVO_4_‐based particles for H_2_O_2_ generation in PS systems primarily focuses on enhancing charge separation and increasing the number of surface active sites. Crystal facet engineering is prominently used to facilitate the spatial separation of photogenerated electrons and holes on the respective {010} and {110} facets of BiVO_4_ particles. Cocatalysts are incorporated on the surface of BiVO_4_ particle to provide active sites to promote the ORR and WOR. In contrast, the development of BiVO_4_‐based photoanodes for H_2_O_2_ generation in PEC systems prioritizes improving the BiVO_4_/electrolyte interfacial reaction kinetics and enhancing selectivity of WOR toward the two‐electron pathway for H_2_O_2_ generation. This is achieved through various surface passivation approaches, such as introducing a passivation layer and tuning of the photoanode's hydrophobicity. Each strategy targets distinct material aspects, including charge separation, surface reaction kinetics, and selectivity. The most successful approach often involves a carefully integration combination of different modification methods to leverage on their synergistic effects and maximize H_2_O_2_ production efficiency.

Moreover, each system requires distinct optimization of reaction conditions for H_2_O_2_ generation (see Table 4). The PS reaction typically operates under O_2_‐saturated conditions as O_2_ serves as the reactant for H_2_O_2_ production via the two‐electron ORR. The temperature of the reaction medium is typically controlled at room temperature or lower to minimize the thermal decomposition of H_2_O_2_. Sacrificial reagents such as methanol or ethanol are also added to expedite hole consumption, minimize charge recombination, and promote H_2_O_2_ production. In contrast, electrolytes containing bicarbonate ion (HCO_3_^−^) are essential for facilitating the direct production of H_2_O_2_ from PEC water oxidation, with CO_2_ bubbling of the electrolyte being beneficial for the operation of BiVO_4_‐based photoanodes.

**Figure 27:**
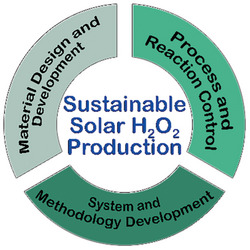
Critical factors in the development of BiVO_4_‐based materials for solar H_2_O_2_ production via PS and PEC systems, pivotal for future industrial applications.

Significant progress has been made in the development of BiVO_4_‐based materials for H_2_O_2_ generation via PS and PEC systems over the past decade. However, this field is still in its nascent stages, and achieving practical applications will require substantial further advancements. **Figure** **27** outlines the key challenges that need be addressed to develop highly efficient BiVO_4_‐based photocatalysts for sustainable H_2_O_2_ production. These challenges are elaborated upon below:

1) *Development of Alternative Cocatalysts*: The current reliance on noble metals such as Pd and Au as cocatalysts in PS systems presents a significant hurdle due to their scarcity and hefty cost. For long‐term sustainability and widespread adoption, it is imperative to explore earth‐abundant and inexpensive alternatives. Additionally, optimizing three‐phase mass transfer is crucial for efficient H_2_O_2_ production via the two‐electron ORR pathway. This entails enhancing O_2_ adsorption onto the catalyst surface and minimizing H_2_O_2_ decomposition by facilitating its desorption to the liquid phase once formed.
2) *Minimization of Sacrificial Reagent Use*: The utilization of sacrificial reagents such as methanol and ethanol in PS systems, while effective in promoting photogenerated electrons for the two‐electron ORR and improving H_2_O_2_ generation, introduces drawbacks. Their use should be minimized due to the potential generation of toxic by‐products and challenges associated with separation of the H_2_O_2_ generated. Alternative strategies for suppressing charge carrier recombination are necessary.
3) *Enhancement of BiVO_4_ Stability*: Understanding of stability of BiVO_4_ in PS and PEC H_2_O_2_ generation is critical for developing efficient and durable solar‐driven H₂O₂ production technologies. While extensive research has explored the chemical and photochemical corrosion mechanisms of BiVO_4_, particularly regarding V dissolution during water splitting, studies specifically addressing its stability in H_2_O_2_ generation are limited. Gaining insights into these photocorrosion mechanisms is essential for devising strategies to enhance BiVO_4_’s stability and optimize its performance in H₂O₂ production.
4) *Understanding and Controlling Competing Reactions*: Solar‐driven H_2_O_2_ production involves competing reactions between H_2_O_2_ formation and decomposition. Current research primarily focuses on demonstrating increased H_2_O_2_ accumulation without a thorough understanding of how different photocatalyst and photoelectrode modification strategies influence reaction mechanisms. Utilizing advanced in‐situ characterization techniques and theoretical calculations can provide valuable insights into the reaction pathways, guiding future advancements.
5) *Achieving Higher H_2_O_2_ Yields*: The current yields of H_2_O_2_ in both PS and PEC systems are insufficient for commercial viability. Future research should prioritize achieving H_2_O_2_ concentrations of at least several tens of millimoles per liter (mmol L^−1^) for practicality, particularly in small‐scale and on‐site applications such as disinfection and bleaching. By focusing on improving H_2_O_2_ yields to meet these concentration targets, researchers can pave way for broader adoption of these technologies.
6) *Standardization of Methodologies and Performance Reporting*: The current disparity in methodologies and performance reporting benchmarks makes it challenging to compare results across different research groups for photocatalytic PS and PEC H_2_O_2_ production studies. Standardization in these aspects is crucial for facilitating easy comparability of their reported performance. Standardized protocols can also serve as a valuable guide for new researchers, enhancing the reliability of research outcomes, ultimately accelerating the growth of this field.
7) *Innovation in Reactor Design*: While batch reactors are currently employed in H_2_O_2_ production via PS and PEC systems, further studies into flow reactors hold promise due to their superior mass transport capabilities. Flow reactors can improve the interaction between reactants and photocatalyst, potentially reducing H_2_O_2_ accumulation near the photocatalyst surface and minimizing its decomposition. Exploring the potential of flow reactors could lead to advancements in H_2_O_2_ production efficiency and process understanding.

Addressing these challenges is essential for advancing the technology toward industrial implementation. This review paper is expected to guide the development of high‐performance photocatalysts, particularly BiVO_4_‐based materials, specifically tailored for PS and PEC systems. By providing a comprehensive overview of the field, it offers a valuable framework for researchers exploring other potential photocatalysts for solar‐driven H_2_O_2_ generation.

## Conflict of Interest

The authors declare no conflict of interest.
